# Quid est *Clea helena?* Evidence for a previously unrecognized radiation of assassin snails (Gastropoda: Buccinoidea: Nassariidae)

**DOI:** 10.7717/peerj.3638

**Published:** 2017-08-11

**Authors:** Ellen E. Strong, Lee Ann Galindo, Yuri I. Kantor

**Affiliations:** 1 Department of Invertebrate Zoology, Smithsonian Institution, National Museum of Natural History, Washington, DC, USA; 2 Institut de Systématique, Évolution, Biodiversité, ISYEB, UMR7205 (CNRS, EPHE, MNHN, UPMC), Muséum National d’Histoire Naturelle, Sorbonne Universités, France; 3 A.N. Severtsov Institute of Ecology and Evolution, Russian Academy of Sciences, Moscow, Russia

**Keywords:** Anatomy, Phylogeny, Biogeography, Freshwater, Invasive species, Systematics, Taxonomy, Biodiversity

## Abstract

The genus *Clea* from SE Asia is from one of only two unrelated families among the megadiverse predatory marine Neogastropoda to have successfully conquered continental waters. While little is known about their anatomy, life history and ecology, interest has grown exponentially in recent years owing to their increasing popularity as aquarium pets. However, the systematic affinities of the genus and the validity of the included species have not been robustly explored. Differences in shell, operculum and radula characters support separation of *Clea* as presently defined into two distinct genera: *Clea*, for the type species *Clea nigricans* and its allies, and *Anentome* for *Clea helena* and allies. A five-gene mitochondrial (COI, 16S, 12S) and nuclear (H3, 28S) gene dataset confirms the placement of *Anentome* as a somewhat isolated offshoot of the family Nassariidae and sister to the estuarine *Nassodonta*. Anatomical data corroborate this grouping and, in conjunction with their phylogenetic placement, support their recognition as a new subfamily, the Anentominae. The assassin snail *Anentome helena*, a popular import through the aquarium trade so named for their voracious appetite for other snails, is found to comprise a complex of at least four species. None of these likely represents true *Anentome helena* described from Java, including a specimen purchased through the aquarium trade under this name in the US and one that was recently found introduced in Singapore, both of which were supported as conspecific with a species from Thailand. The introduction of *Anentome* “*helena*” through the aquarium trade constitutes a significant threat to native aquatic snail faunas which are often already highly imperiled. Comprehensive systematic revision of this previously unrecognized species complex is urgently needed to facilitate communication and manage this emerging threat.

## Introduction

The Neogastropoda is a very large and successful clade of primarily marine predatory caenogastropods with Cretaceous origins that diversified rapidly during the Cenozoic (Ponder, 1973; Vermeij, 1977; Taylor, Morris & Taylor, 1980). Although a number of species are found in areas with decreased and/or fluctuating salinity such as estuaries and the coastal reaches of large rivers, very few species have conquered continental waters. These include species in only two genera from two distantly related families: two species in the genus *Rivomarginella* Brandt, 1968 (Marginellidae) from rivers, lakes and canals in SE Asia (Brandt, 1968) and a number of species currently united in the genus *Clea* H. Adams & A. Adams, 1855.

**Figure 1:**
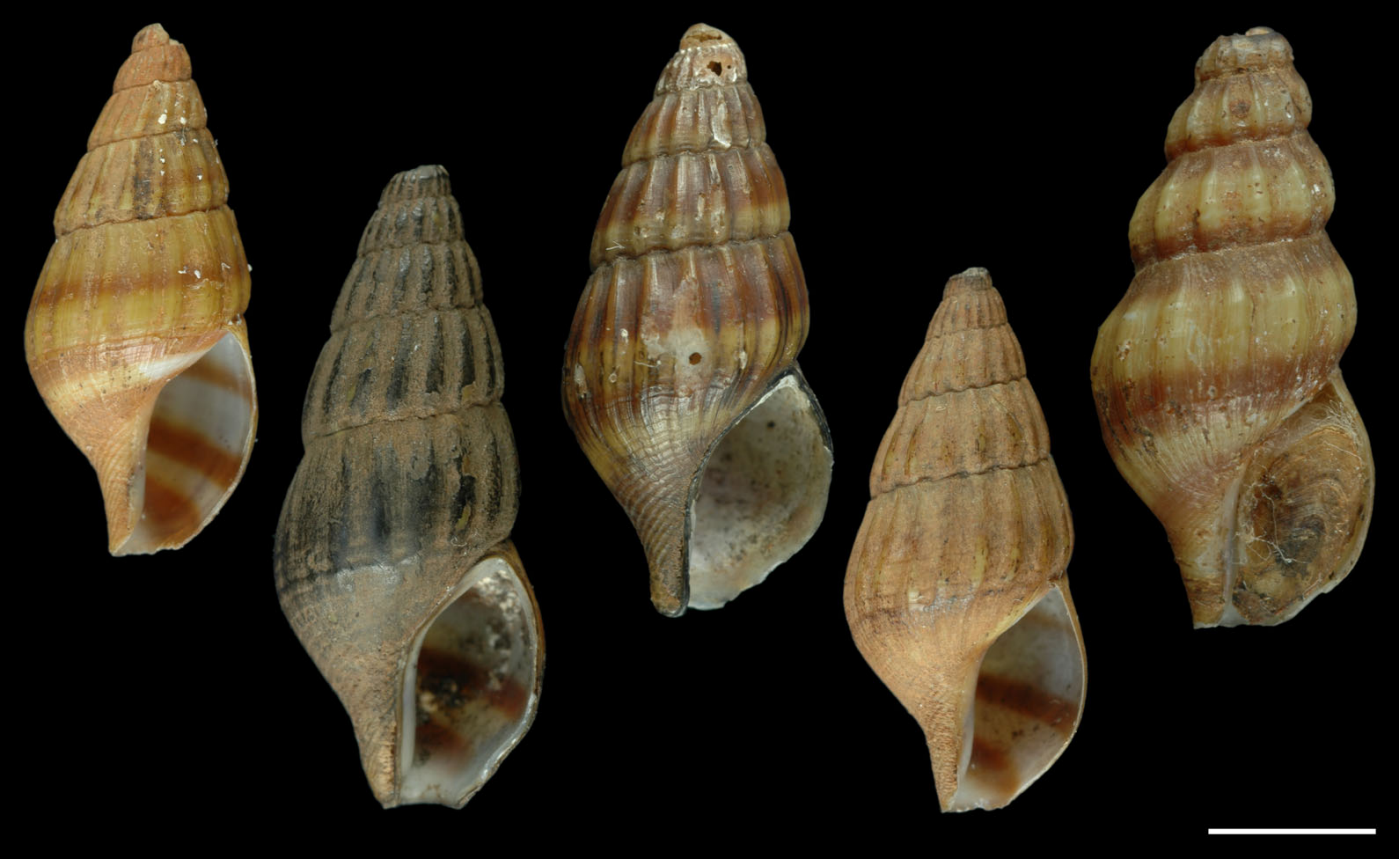
Paralectotypes of *Melania helena* von dem Busch, 1847. MNHN IM-2000-27679. Scale bar: 5 mm.

Members of the latter genus are found primarily in the broad lower reaches of coastal rivers, as well as lakes and ponds, and are distributed in southern China and throughout Southeast Asia around the Sunda Shelf in southwestern Philippines, Borneo, Java, Sumatra, peninsular Malaysia, Thailand, Laos, Cambodia and Vietnam (van Benthem Jutting, 1929, 1959; Brandt, 1974; Liu, Wang & Zhang, 1980). Among these, *Clea helena* (von dem Busch, 1847) (Fig. 1), sometimes referred to *Anentome* at the rank of genus or subgenus, has recently become a popular commodity in the ornamental pet industry (Ng et al., 2016a, 2016b). The ability of this species to tolerate still waters (Brandt, 1974) has been a significant factor in their adaptability to captive conditions (Coelho, Dinis & Reis, 2013). Commonly known as “assassin snails” among other names for their voracious appetite for other gastropods, they are non-selective predators and scavengers of a wide variety of gastropods, including those larger than themselves, but may also feed on fish eggs and shrimp (Bogan & Hanneman, 2013). There is increasing concern over the threat they pose to native aquatic snail populations should they be introduced outside their natural range (Mienis, 2011; Bogan & Hanneman, 2013; Ng et al., 2016a).

Little is known about the anatomy and biology of *Clea*, with much of what is to be found in the published record relating primarily to taxonomy and distribution, although interest in the life history and ecology of the genus has grown with their increasing popularity as aquarium pets (Monks, 2009; SiputKuning Journal, 2010; Coelho, Dinis & Reis, 2013). In addition, *Clea nigricans* A. Adams, 1855 has been used to explore the potential impact of climate change on tropical freshwater systems (Polgar et al., 2015), and *Clea helena* has been touted as a possible model for developmental and environmental physiology (Newel & Bourne, 2013). Given the burgeoning interest in these species across diverse fields, a solid systematic foundation is a fundamental need yet neither the affinities of the genus nor the validity of the included species and the names that should be applied to them have been robustly explored.

*Clea* has a complicated taxonomic history. With 30 nominal species, of which 15 are currently recognized as valid (MolluscaBase, 2017), its members have been referred to diverse caenogastropod families, including the cerithioidean families “Melaniidae” (an invalid name for Thiaridae), Melanopsidae, or Planaxidae (Adams & Adams, 1858; Reeve, 1860; Brot, 1862, 1868; Crosse, 1886; Cossmann, 1909; Thiele, 1929; Wenz, 1940), and until recently were united in the neogastropod family Buccinidae (see Galindo et al., 2016). A comprehensive reassessment of the phylogeny and systematics of the Nassariidae (Buccinoidea) supported the placement of a single sequenced representative of *Clea* as an isolated branch in a redefined and expanded concept of the family (Galindo et al., 2016).

Most of the nominal species of *Clea* were described during the mid to late 1800s based primarily on (sometimes minor) differences in shell morphology. Beginning with the work of Brot (1881), authors began to embrace a broader, more variable species concept; for example, Smith (1895a), who enumerated six varieties of *Clea nigricans*, commented on the “enormous” conchological differences between some of them, but stated that he preferred to not recognize them as distinct species, pending further evidence “than is furnished by the shells” (1895a: 252). The most recent review is Brandt’s (1974) work on the Thai freshwater fauna. Brandt (1974) synonymized seven species with *Clea helena* based on purely conchological grounds, thereby establishing a broad definition of the species, “…extremely variable with regard to size, shape and costulation” (1974: 202).

Understanding the conchological basis for the recognition of species can only be appreciated within the context of a robust systematic framework, which heretofore has been lacking. Here we use a multi-gene mitochondrial and nuclear dataset, expanded taxonomic sampling of members of the genus, and anatomical data, to confirm its systematic affinities and to gain insight into the origins and diversity of this clade of freshwater predators.

## Materials and Methods

### Anatomical investigations

**Figure 2:**
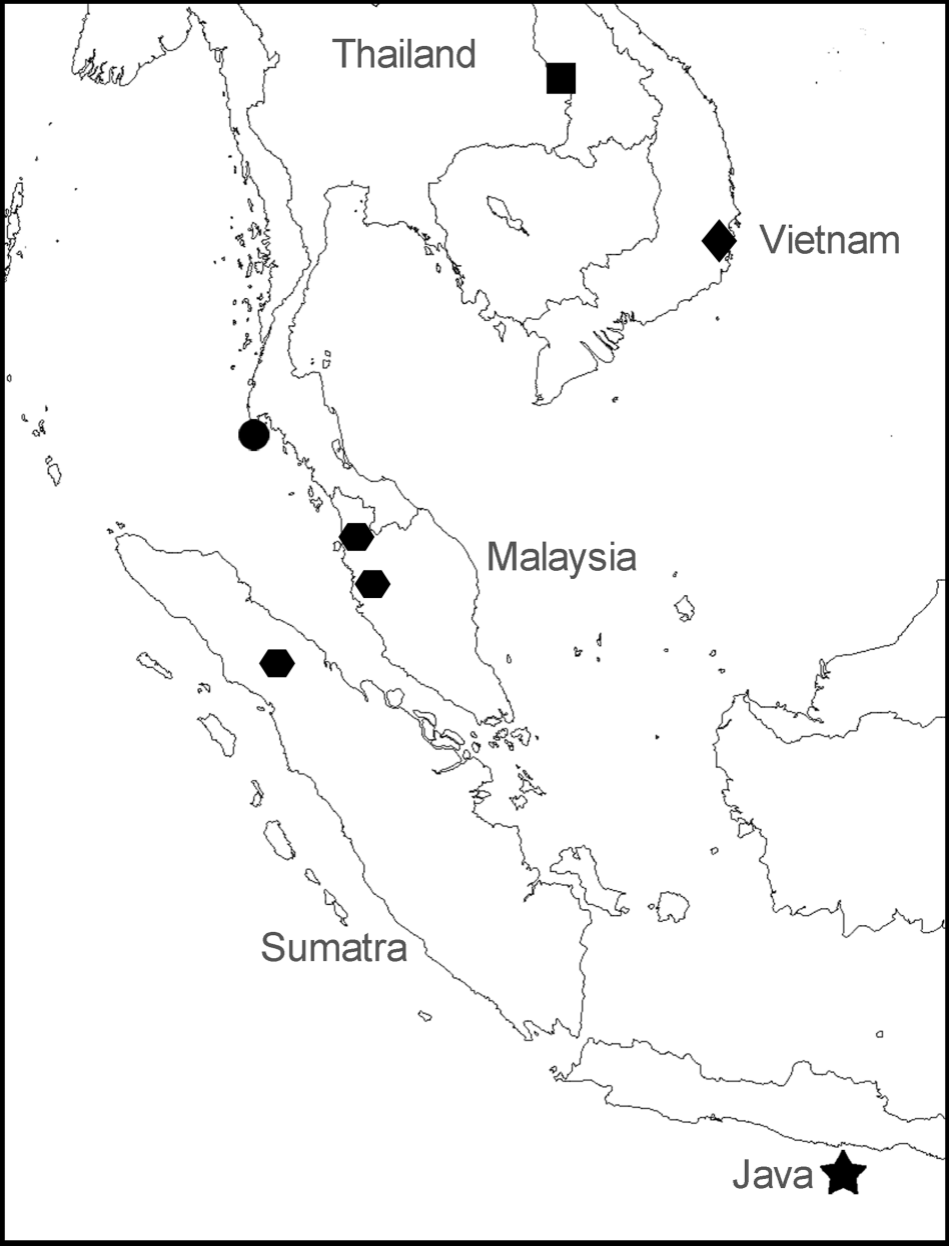
Sampling locations for specimens of *Anentome* “*helena*.” •, species A. ▪, species B. 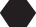, species C. ♦, species D. ★, type locality of *Melania helena* on “Java.” See Table 1 for details.

Specimens identified as *Clea helena* were assembled from Vietnam, Thailand, Sumatra and peninsular Malaysia (Fig. 2); one sample was purchased through the aquarium trade in Washington, DC. Specimens of *Nassodonta dorri* (Wattebled, 1886) from Vietnam were used for anatomical comparisons. Bodies of animals for the anatomical investigations were separated by cracking the shells with a vice. Tissues of sequenced specimens were fixed in 95–98% ethanol; shells of sequenced specimens were kept intact, dried and registered as vouchers. Specimens were dissected under a Leica MZ 6 or MZ 16.5 stereo microscope and stained with toluidine blue to enhance contrast. Radulae were mounted on glass cover slips, glued to aluminum stubs with carbon adhesive tabs, coated with gold and examined with a JEOL JSM5410LV Low Vacuum Scanning Microscope at the Centre de Recherche sur la Conservation des Collections in Paris (MNHN-MCC-CNRS USR 3224) and with a Tescan TS5130MM Scanning Electron Microscope at the A.N. Severtsov Institute in Moscow.

### Molecular and phylogenetic analyses

The taxonomic sampling scheme used herein included 23 individuals tentatively identified as *Clea helena*, six specimens of *Nassodonta dorri*, and a reduced subset of 27 taxa used in the comprehensive phylogenetic analysis of the Nassariidae of Galindo et al. (2016). Six representatives from other families among the Buccinoidea were also included and the tree was rooted with *Cancellaria cooperii* Gabb, 1865 (Cancellariidae, Cancellarioidea).

Molecular sequencing was carried out at the Service de Systématique Moléculaire (MNHN-Paris VI UMR 7138 UMS 2700). Partial COI, 16S, 12S, 28S (partial C1 and D2 domains) and H3 genes were amplified, using the primers of Galindo et al. (2016), Palumbi et al. (1991), Simon, Franke & Martin (1991), Chisholm et al. (2001) and Colgan et al. (1998), respectively. PCR reactions were performed in 20 μl volumes, containing between 1 and 2 μl of genomic DNA, 1× reaction buffer, 2.5 mM MgCl_2_, 0.26 mM dNTP, 0.3 μM of each primer, 5% DMSO and 5% BSA (10 mg/l) and 1.5 units of Q-Bio Taq (QBiogene, Carlsbad, CA, USA). Annealing temperature was 54 °C for 40 s for COI, 53 °C×35 s for 16S, 63 °C × 40 s for 12S, 58 °C × 40 s for 28S and 55 °C × 35 s for H3. Bidirectional sequencing was carried out by the Centre National de Séquençage (Genoscope, Essonne, France). Chromatograms were visually inspected and edited as necessary in CodonCode Aligner 4.0.4 (CodonCode Corporation, Dedham, MA, USA), and aligned with Clustal W (Larkin et al., 2007) as implemented in MEGA5 (Tamura et al., 2011). COI was translated into amino acids to check for stop codons and frameshift mutations. All newly generated sequences have been deposited in GenBank (Table 1).

**Table 1:** Voucher registration number, locality, and GenBank accession numbers of analyzed specimens.

Voucher	Species	Locality	Accession numbers				
			COI	16S	12S	28S	H3
*Outgroups*							
MZURBAU00797	*Cancellaria cooperii* Gabb, 1865		FM999156	FM999104	FM999073	FM999135	–
MNHN IM 2009-18853	*Belomitra paschalis* (Thiele, 1925)		JQ950229	JQ950147	–	JQ950188	–
LSGB2341301	*Fusinus colus* (Linnaeus, 1758)		HQ834100	HQ833955	HQ833907	–	HQ834178
LSGB233031	*Hemifusus ternatanus* (Gmelin, 1791)		HM180609	JN052949	HQ833889	–	HQ834160
MZURBAU00698	*Pisania striata* (Gmelin, 1791)		FM999175	FM999128	FM999097	–	–
	*Neptunea antiqua* (Linnaeus, 1758)		AF373886	GQ290496	GQ290514	GQ290567	GQ290635
	*Buccinum undatum* (Linnaeus, 1758)		EF528303	FN677455	FN677400	FN677456	–
*Anentominae*							
**MNHN IM 2013-52178**	***Anentome* sp. A**	**Aquarium trade US**	**KY773629**	**KY706403**	**–**	**KY706441**	**KY706457**
**MNHN IM 2013-52183**	***Anentome* sp. A**	**FW Pond in Phuket Park Thailand**	**KY773633**	**KY706406**	**–**	**–**	**–**
**MNHN IM 2013-52184**	***Anentome* sp. A**	**FW Pond in Phuket Park Thailand**	**KY773634**	**KY706407**	**–**	**KY706444**	**KY706461**
**MNHN IM 2013-52185**	***Anentome* sp. A**	**FW Pond in Phuket Park Thailand**	**KY773635**	**KY706408**	**–**	**KY706445**	**KY706462**
**MNHN IM 2013-52175**	***Anentome* sp. B**	**Sam Phan Bok, Pho Sai District, Ubon Ratchathani, NE Thailand, Mekong River**	**KY773627**	**KY706400**	**KY706424**	**KY706438**	**KY706454**
**MNHN IM 2013-52176**	***Anentome* sp. B**	**Sam Phan Bok, Pho Sai District, Ubon Ratchathani, NE Thailand, Mekong River**	**KY773628**	**KY706401**	**KY706425**	**KY706439**	**KY706455**
**MNHN IM 2013-52177**	***Anentome* sp. B**	**Sam Phan Bok, Pho Sai District, Ubon Ratchathani, NE Thailand, Mekong River**	**–**	**KY706402**	**–**	**KY706440**	**KY706456**
**MNHN IM 2013-52179**	***Anentome* sp. C**	**Kellie’s Castle. Malaysia, 4°28′30.9″N, 101°05′14.4″E**	**KY773630**	**–**	**–**	**–**	**KY706458**
**MNHN IM 2013-52180**	***Anentome* sp. C**	**Temenggor Lake. Malaysia**	**KY773631**	**KY706404**	**–**	**KY706442**	**KY706459**
**MNHN IM 2013-52181**	***Anentome* sp. C**	**Temenggor Lake. Malaysia**	**KY773632**	**KY706405**	**–**	**KY706443**	**KY706460**
**MNHN IM 2013-59228**	***Anentome* sp. C**	**Toba Lake, N. Sumatra 2°36′8N, 98°55′2″E**	**KY773636**	**KY706409**	**KY706426**	**–**	**–**
**MNHN IM 2013-59229**	***Anentome* sp. C**	**Toba Lake, N. Sumatra 2°36′8N, 98°55′2″E**	**–**	**KY706410**	**KY706427**	**KY706446**	**–**
**MNHN IM 2013-59230**	***Anentome* sp. C**	**Toba Lake, N. Sumatra 2°36′8N, 98°55′2″E**	**KY773637**	**KY706411**	**KY706428**	**KY706447**	**KY706463**
**MNHN IM 2013-59231**	***Anentome* sp. C**	**Toba Lake, N. Sumatra 2°36′8N, 98°55′2″E**	**–**	**KY887752**	**KY887755**	**KY706448**	**–**
**MNHN IM 2013-59232**	***Anentome* sp. C**	**Toba Lake, N. Sumatra 2°36′8N, 98°55′2″E**	**–**	**KY887753**	**KY887756**	**KY706449**	**–**
**MNHN IM 2013-59233**	***Anentome* sp. C**	**Toba Lake, N. Sumatra 2°36′8N, 98°55′2″E**	**–**	**KY887754**	**KY887757**	**KY706450**	**–**
**MNHN IM 2013-59234**	***Anentome* sp. C**	**Toba Lake, N. Sumatra 2°36′8N, 98°55′2″E**	**–**	**KY706412**	**KY706429**	**KY706451**	**–**
**MNHN IM 2009-29657**	***Anentome* sp. D**	**Kai-River, Vietnam 12°16.7′N, 108°59.57′E**	**–**	**KY706397**	**KY706419**	**–**	**–**
MNHN IM 2009-29658	*Anentome* sp. D	Kai-River, Vietnam 12°16.7′N, 108°59.57′E	KY451412	KY488922	KY489121	KY489289	KY489374
**MNHN IM 2009-29659**	***Anentome* sp. D**	**Kai-River, Vietnam 12°16.7′N, 108°59.57′E**	**–**	**–**	**KY706420**	**KY706435**	**–**
**MNHN IM 2009-29660**	***Anentome* sp. D**	**Kai-River, Vietnam 12°16.7′N, 108°59.57′E**	**–**	**KY706398**	**KY706421**	**–**	**–**
**MNHN IM 2009-29661**	***Anentome* sp. D**	**Kai-River, Vietnam 12°16.7′N, 108°59.57′E**	**KY773626**	**KY706399**	**KY706422**	**KY706436**	**KY706453**
**MNHN IM 2009-29663**	***Anentome* sp. D**	**Kai-River, Vietnam 12°16.7′N, 108°59.57′E**	**–**	**–**	**KY706423**	**KY706437**	**–**
**MNHN IM 2009-20638**	***Nassodonta dorri* (Wattebled, 1886)**	**Province Binh Thuan, Phan Ri River Song Ni, 11°10.57′N, 108°33.70′E Vietnam**	**KY773620**	**KY706391**	**KY706413**	**KY706430**	**KY706452**
**MNHN IM 2009-20640**	***Nassodonta dorri* (Wattebled, 1886)**	**Province Binh Thuan, Phan Ri River Song Ni, 11°10.57′N, 108°33.70′E Vietnam**	**KY773621**	**KY706392**	**KY706414**	**KY706431**	**–**
**MNHN IM 2009-20642**	***Nassodonta dorri* (Wattebled, 1886)**	**Province Binh Thuan, Phan Ri River Song Ni, 11°10.57′N, 108°33.70′E Vietnam**	**KY773622**	**KY706393**	**KY706415**	**KY706432**	**–**
**MNHN IM 2009-20643**	***Nassodonta dorri* (Wattebled, 1886)**	**Province Binh Thuan, Phan Ri River Song Ni, 11°10.57′N, 108°33.70′E Vietnam**	**KY773623**	**KY706394**	**KY706416**	**KY706433**	**–**
**MNHN IM 2009-20644**	***Nassodonta dorri* (Wattebled, 1886)**	**Province Binh Thuan, Phan Ri River Song Ni, 11°10.57′N, 108°33.70′E Vietnam**	**KY773624**	**KY706395**	**KY706417**	**KY706434**	**–**
**MNHN IM 2009-20653**	***Nassodonta dorri* (Wattebled, 1886)**	**Province Binh Thuan, Phan Ri River Song Ni, 11°10.57′N, 108°33.70′E Vietnam**	**KY773625**	**KY706396**	**KY706418**	**–**	**–**
*Buccinanopsinae*							
MZUSP108269	*Buccinanops cochlidium* (Dillwyn, 1817)	Santos Municipality, São Paulo, Brazil, 24°05.70′S, 46°20.07′W	KY451221	KY488731	KY488928	KY489126	KY489295
MNHN IM 2009-24004	*Buccinanops deformis* (King, 1832)	Puerto Madryn, Argentina	KY451220	KY488730	KY488927	KY489125	KY489294
*Bulliinae*							
MNHN IM 2009-22716	*Bullia cataphracta* Kilburn, 1978	Maputo Bay, Mozambique, 25°58.2′S 32°59.4′E	KY451223	KY488732	KY488929	–	KY489297
MNHN IM 2009-22535	*Bullia diluta* (Krauss, 1848)	Maputo Bay, Mozambique, 25°58.2′S 32°59.4′E	KY451224	KY488733	KY488930	–	KY489298
*Cylleninae*							
MNHN IM 2009-12765	*Cyllene parvula* Bozzetti, 2014	Cap Malaimpioka, Madagascar, 25°21.3-6′S 44°44.6-9′E	KY451237	KY488742	KY488942	KY489132	KY489309
MNHN IM 2007-31755	*Cyllene pulchella* Adams & Reeve, 1850	West Tangoa Island, Vanuatu, 15°35.4′S 166°58.7′E	KY451238	KY488743	KY488943	KY489133	KY489310
	*Nassaria magnifica* Lischke, 1871	Japan	FJ712703	AB044264	–	FJ710100	–
MNHN IM 2009-13155	*Nassaria* sp.	Vitiaz Strait, Papua New Guinea, 06°03′S 147°36′E	KY451414	KY488924	KY489123	KY489290	KY489376
MNHN IM 2013-52188	*Tomlinia frausseni* Thach, 2014	Ho Chi Minh City, Vietnam	KY451417	KY488926	–	–	KY489378
*Photinae*							
MNHN IM 2013-8450	*Antillophos candeanus* (d′Orbigny, 1842)	Petit cul de sac Marin, Guadeloupe 16°13.41′N 61°31.83′W	KY451407	KY488917	–	KY489286	KY489372
MNHN IM 2009-24414	*Engoniophos unicinctus* (Say, 1826)	Anse Basin (Morne-à-l′eau), Guadeloupe, 16°20.45′N, 61°31.55′W	KY451413	KY488923	KY489122	–	KY489375
MNHN IM 2009-20613	*Phos alabastrum* Fraussen, 2003	Yaté Pass, New Caledonia, 22°06′S 167°04′E	KY451405	KY488914	KY489115	KY489284	KY489373
MNHN IM 2009-13112	*Phos hirasei* Sowerby III, 1913	Jacquinot Bay, New Brittany, Papua-New Guinea, 05°34′S 151°32′E	KY451410	KY488920	KY489119	KY489288	–
MNHN IM 2009-13144	*Phos* cf. *hirasei*	Manus Island, Papua New Guinea, 02°10′S 147°15′E	KY451408	KY488918	KY489117	KY489287	–
LSGB232091	*Phos senticosus* (Linnaeus, 1758)	China	JN053008	JN052944	HQ833884	–	HQ834155
*Dorsaninae*							
MNHN IM 2013-52428	*Dorsanum miran* (Bruguière, 1789)	Mauritania	KY451239	KY488744	KY488944	KY489134	KY489311
*Nassariinae*							
MNHN IM 2007-31898	*Nassarius arcularia* (Linnaeus, 1758)	Panglao Island, inside lagoon near Doljo Pt, Philippines, 9°35.1′N 123°43.6′E	KY451259	KY488766	KY488968	KY489155	KY489317
MNHN IM 2009-21554	*Nassarius boucheti* Kool 2004	Ounia Pass, New Caledonia, 21°52′S 166°51′E	KY451266	KY488772	KY488975	KY489161	–
MNHN IM 2009-29668	*Nassarius conoidalis* (Deshayes in Belanger, 1832)	Vietnam, 12°10.44′N 109°16.30′E	KY451284	KY488790	KY488992	KY489176	–
MNHN IM 2007-31730	*Nassarius niger* (Hombron & Jacquinot, 1848)	Nasouli River, Vanuatu, 15°34.8′S 167°01.6′E	KY451241	KY488746	KY488946	KY489136	KY489313
MNHN IM 2007-31729	*Nassarius radians* Kool & Galindo 2014	N Urélapa Island, Vanuatu, 15°35.9′S–15°36.0′S 167°01.3/01.6′E	KC970058	KY488864	KY489065	KY489240	KY489353
MNHN IM 2009-23946	*Naytia glabrata* (G. B. Sowerby II, 1842)	Congo	KY451307	KY488812	KY489014	KY489191	KY489332
MNHN IM 2009-23948	*Naytia granulosa* (Lamarck, 1822)	Port Zanaga, Congo, 04°43.28′S 11°48.63′E	KY451225	KY488734	KY488931	KY489128	KY489299
MNHN IM 2009-24320	*Phrontis antillarum* (d′Orbigny 1847)	Port-Louis, Guadeloupe, 16°23.26′N 61°31.79′W	KY451258	KY488765	KY488967	KY489154	KY489316
MNHN IM 2009-24334	*Phrontis vibex* (Say, 1822)	Banc Frotte-ton-cul, Guadeloupe, 16°17.35′N 61°34.74′W	KY451402	KY488911	KY489112	KY489281	–
MNHN IM 2009-21755	*Tritia obsoleta* (Say, 1822)	Charleston, South Carolina, USA	KY451244	KY488748	KY488949	KY489139	KY489315
MNHN IM 2009-22330	*Tritia reticulata* (Linnaeus, 1758)	Agaete, Gran Canaria, Spain	KY451356	KY488865	KY489067	KY489242	KY489354

**Note:**

Newly generated sequences shown in bold. All other nassariid sequences, with the exception of *Nassaria magnifica* and *Phos senticosus*, are from Galindo et al. (2016). Outgroup sequences were downloaded from GenBank. Generic classification follows that proposed herein.

PartitionFinder 1.0 (Lanfear et al., 2012) was used to select the best-fit partitioning schemes and models for phylogenetic analysis, which favored two partitions (first COI codon position, 12S, 16S vs second and third COI codon positions, 28S, and H3) and the GTR+I+G substitution model for both partitions. In the absence of significant incongruence, Bayesian phylogenies for three datasets (mitochondrial, nuclear and concatenated datasets) were each inferred using MrBayes 3.1.6 (Ronquist & Huelsenbeck, 2003) as implemented on the CIPRES Science Gateway (Miller, Pfeiffer & Schwartz, 2010). Bayesian analyses, consisting of two independent replicates with eight chains each were run for 60,000,000 Markov chain Monte Carlo (MCMC) generations with a sampling frequency of one tree every 100 generations. MCMC convergence, likelihood curves and standard deviation of split frequencies were assessed using Tracer 1.6 (Rambaut et al., 2014). The first 25% trees were discarded as burn-in and a 50% majority rule consensus tree constructed. Vouchers from the molecular and anatomical investigations have been deposited in the collections of the Muséum national d’Histoire naturelle in Paris (MNHN) and the National Museum of Natural History in Washington, D.C. (USNM) (see Table 1).

### Nomenclatural acts

The electronic version of this article in portable document format (PDF) will represent a published work according to the International Commission on Zoological Nomenclature (ICZN), and hence the new names contained in the electronic version are effectively published under that code from the electronic edition alone. This published work and the nomenclatural acts it contains have been registered in ZooBank, the online registration system for the ICZN. The ZooBank LSIDs (Life Science Identifiers) can be resolved and the associated information viewed through any standard web browser by appending the LSID to the prefix http://zoobank.org/. The LSID for this publication is: urn:lsid:zoobank.org:pub:C4D12ABC-3DE3-429B-8207-05D549989DD5. The LSID for the new subfamily Anentominae is: urn:lsid:zoobank.org:pub:C4D12ABC-3DE3-429B-8207-05D549989DD5. The online version of this work is archived and available from the following digital repositories: PeerJ, PubMed Central and CLOCKSS.

## Results

### Molecular analyses

**Figure 3:**
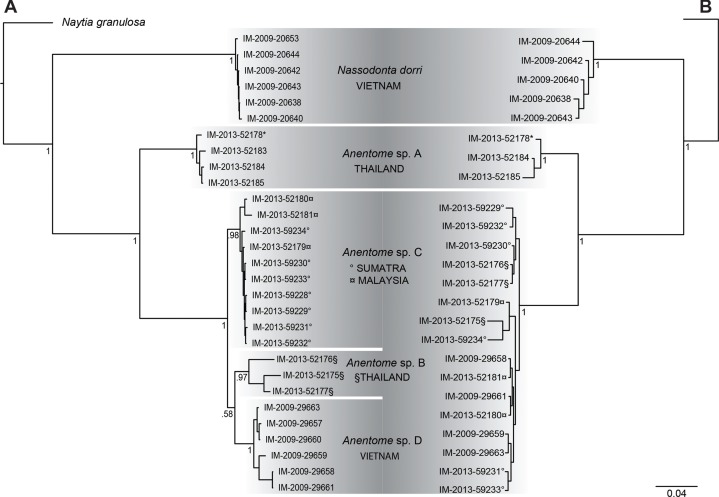
Bayesian phylograms based on separate analyses of the mitochondrial and nuclear gene datasets. (A) Phylogram based on the mitochondrial gene dataset (COI, 12S, 16S). (B) Phylogram based on the nuclear gene dataset (28S, H3). Bayesian posterior probabilities are indicated at the nodes; values ≥0.95 were considered significant. “*” indicates sequenced aquarium-trade voucher. Generic classification follows that proposed herein. See Table 1 for sources. Scale bar indicates number of nucleotide substitutions per site.

Results of Bayesian analysis (Fig. 3A) of the mitochondrial dataset [partial COI (13 individuals), 16S (20 individuals) and 12S (15 individuals) sequences (see Table 1)] recovered the aquarium specimen within a clade with specimens from Phuket, Thailand (*Anentome* species A; classification follows that proposed herein, see Systematics for details) with high support (PP = 1). In Bayesian analysis of the COI data alone (results not shown), the sequenced voucher of the species introduced to Singapore (Ng et al., 2016b) is also conspecific with species A, and is separated from the individuals in this clade by 0.025–0.032 uncorrected pairwise sequence divergence. Species B, also from Thailand, species C from Sumatra and Malaysia, and species D from Vietnam, were all recovered as monophyletic with high support (PP ≥ 0.97). Species B and species D were supported as sister taxa, but with no support (PP = 0.58). The distribution of Kimura-corrected average pairwise distances in COI typically were low within clades and ranged from 0.000 to 0.012, with the exception of species B with 0.080, which is more representative of the distances seen between clades (see Discussion). Results of Bayesian analysis of the nuclear gene dataset (28S, H3) (Fig. 3B) were characterized by short internodal distances, with only species A supported as monophyletic with high support (PP = 1). All other individuals were united in a single clade with high support (PP = 1), but with no support for relationships among them, reflecting the more highly conserved nature of the nuclear genes, and their greater utility for resolving relationships above the species level.

**Figure 4:**
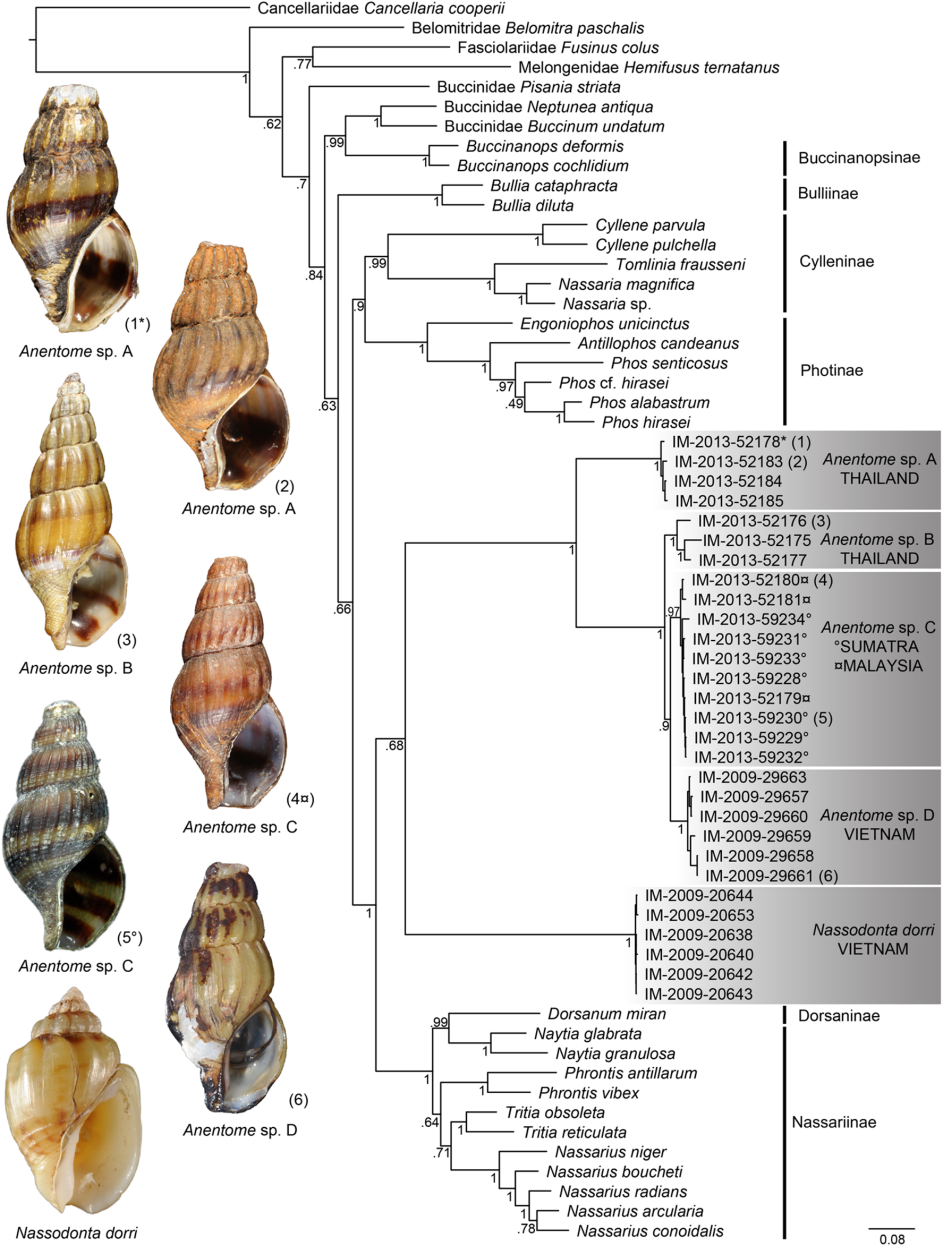
Bayesian phylogram based on a concatenated mitochondrial (COI, 12S, 16S) and nuclear gene (28S, H3) dataset. Bayesian posterior probabilities are indicated at the nodes; values ≥0.95 were considered significant. A sequenced voucher is figured for each clade. “*” indicates sequenced aquarium-trade voucher. Generic classification follows that proposed herein. See Table 1 for sources. Scale bar indicates number of nucleotide substitutions per site.

Bayesian analysis (Fig. 4) of the concatenated mitochondrial and nuclear gene dataset (COI, 16S, 12S, 28S, H3) with a total aligned length of 2927 bp for 63 terminals, including 23 *Anentome* and six *Nassodonta* individuals, supported the main groupings identified by Galindo et al. (2016), but perhaps unsurprisingly given the severe subsampling of the present analysis, not the relationships among them. Nevertheless, the placement of *Anentome* as a somewhat isolated offshoot of the Nassariidae is confirmed, as sister to a large clade uniting the Dorsaninae and Nassariinae. In contrast to the results of Galindo et al. (2016) which supported the single sequenced individuals of *Anentome* (= *Anentome* sp. D) and *Nassodonta* as independent branches, the expanded sampling here yielded a sister group relationship between the two albeit without significant support (PP = 0.68).

Relationships among the four lineages of *Anentome* “*helena*” were resolved with high support (PP = 1), with the exception of the sister group relationship between *Anentome* sp. C (Sumatra, Malaysia) and sp. D (Vietnam) (PP = 0.90) which was not recovered in the analysis of the mitochondrial gene dataset. All species were supported as monophyletic with PP = 1 except species C from Sumatra and Malaysia (PP = 0.97). As in the mitochondrial gene tree, a lineage from Thailand (species A) included the specimen purchased through the aquarium trade.

### Anatomy

*Anentome* sp. A

Material examined: Specimens purchased through aquarium trade, Washington DC, (USNM 1405293); freshwater pond, Phuket, Thailand, July 2010 (MNHN uncataloged).

**Figure 5:**
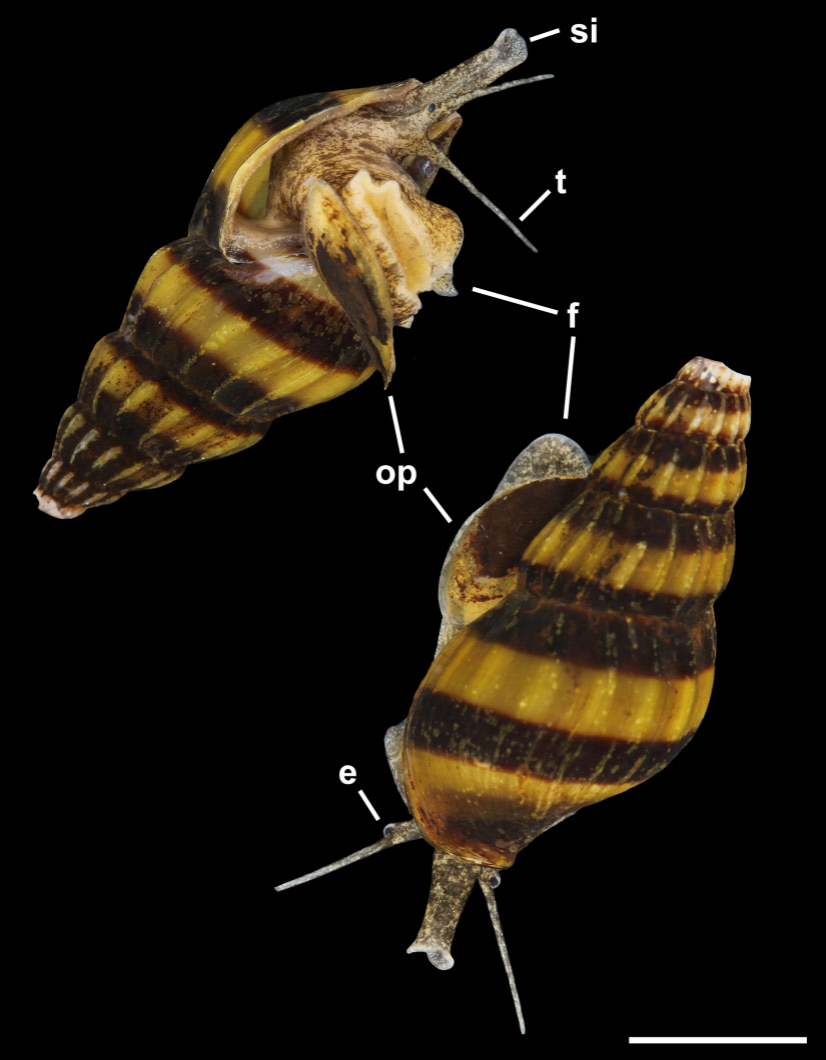
Living animal of *Anentome* sp. A. Apertural and abapertural views. Aquarium-trade specimen. e, eye; f, foot; op, operculum; si, siphon; t, cephalic tentacle. Scale bar: 5 mm.

**Figure 6:**
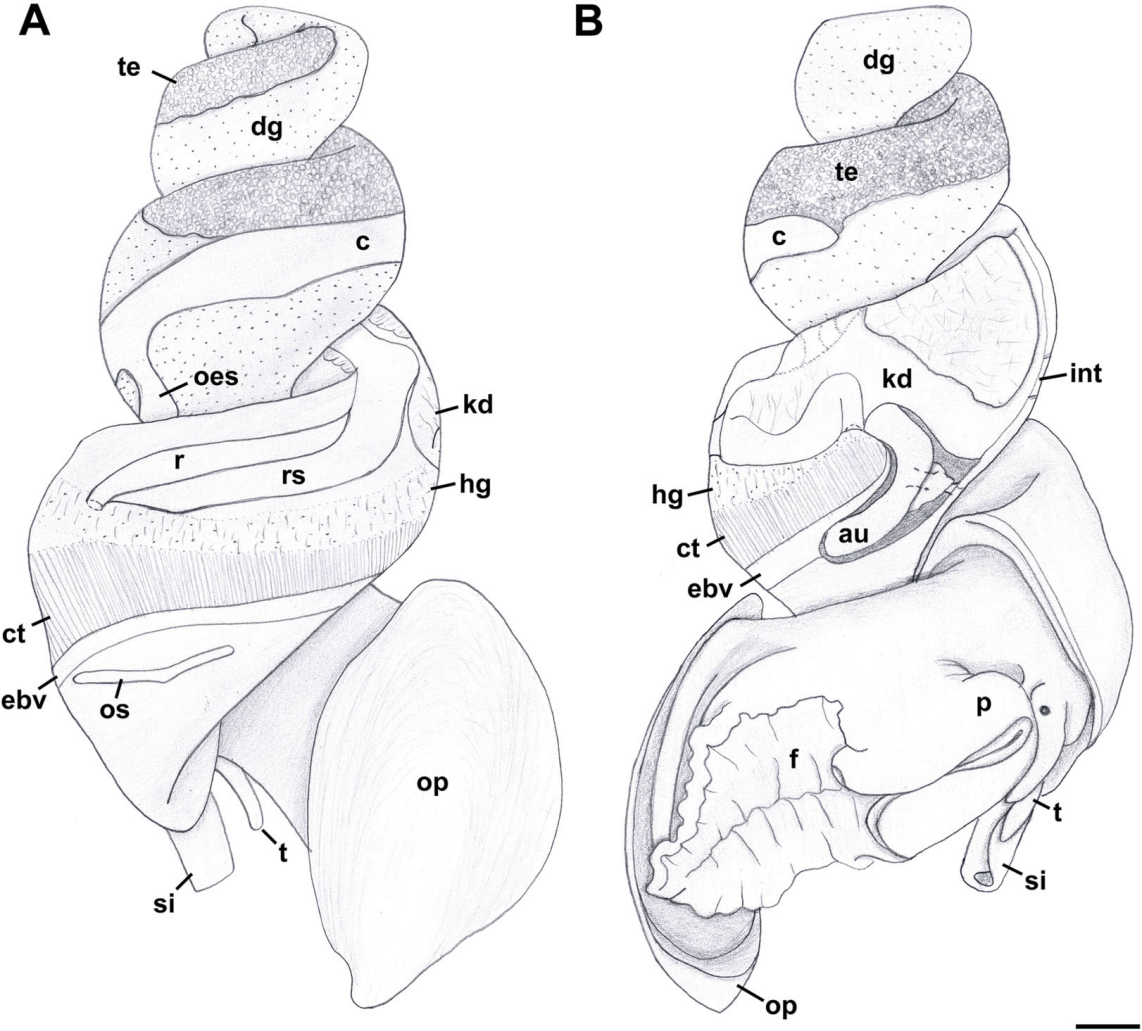
External anatomy of *Anentome* sp. A. (A) Apertural view. (B) Abapertural view. au, auricle; c, caecum; ct, ctenidium; dg, digestive gland; ebv, efferent branchial vein; f, foot; hg, hypobranchial gland; int, intestine; kd, kidney; oes, esophagus; op, operculum; os, osphradium; p, propodium; r, rectum; rs, rectal sinus; si, siphon; t, cephalic tentacle; te, testis. Scale bar: 1 mm.

**Figure 7:**
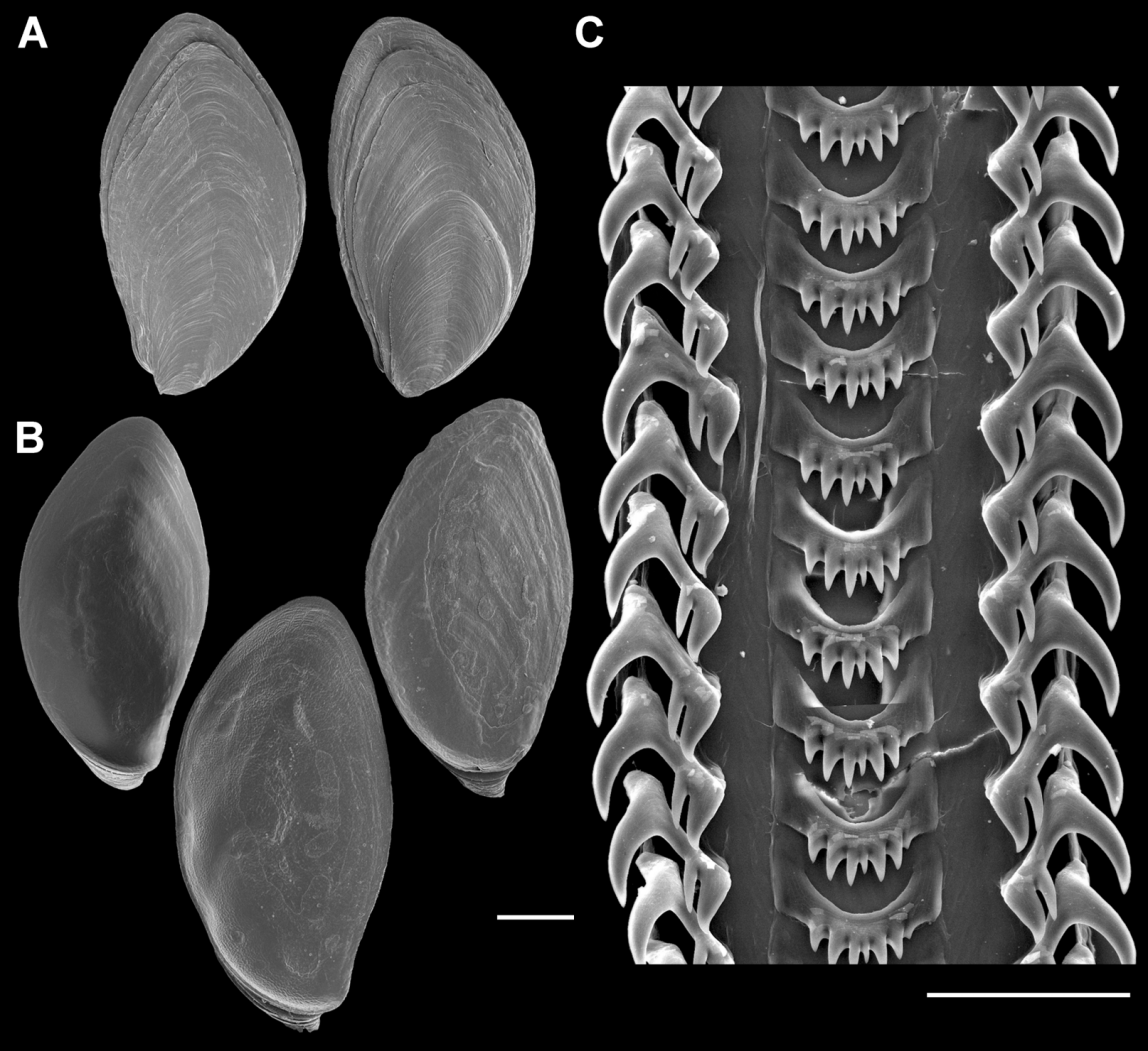
Operculum and radula morphology of *Anentome* sp. A. (A) External, and (B) internal views of operculae. Scale bars: (A, B) 1 mm, (C) 100 μm.

External Anatomy: Mature animal comprising roughly 3.5 whorls in preserved specimens. Animal light yellow, with dense pattern of irregular black blotches covering external surface of headfoot; black blotches fewer and more dispersed on foot sole. Mantle margin, internal surface of siphon and external surface of siphon along seam marked with small, whitish granules (Fig. 5). Head small with long, slender cephalic tentacles (Fig. 5: t), roughly half of siphon in length in preserved specimens, with eyes slightly elevated on prominent ocular peduncles at tentacle outer bases. Foot narrowly oval with small attachment disc to operculum (Fig. 5: op). Prominent propodium separated from mesopodium by conspicuous constriction, and with very deep propodial pedal gland along anterior edge, lined with two histologically distinct subepithelial gland cells. Short, deep transverse ovipositor short distance behind front edge of foot. Metapodium lacking posterior tentacles. Operculum large, spanning nearly entire aperture, elongate oval, concave, with basal nucleus slightly turned to left, and thickened, pointed, elevated process behind (Figs. 6A, 7A and 7B). Mantle cavity long, roughly one whorl in length, posteriorly bounded by reno-pericardial complex (Fig. 6). Mantle cavity markedly asymmetrical, deeper along left side in front of pericardium. Columellar muscle thick, simple, not subdivided, spanning roughly one whorl from attachment at rear of mantle cavity to operculum. Ctenidium (Fig. 6: ct) extending along left side of mantle cavity from near mantle edge to narrow, posterior base of mantle cavity at left; anterior tip of ctenidium slightly curving to end adjacent to more anterior siphonal flap. Long siphon (Fig. 6: si) emerging from behind left anterior mantle edge in front of gill, to left of head. Short, narrow osphradium lying to left of anterior ctenidium, at base of siphon, slightly posterior to curved anterior tip of ctenidium. Osphradium bipectinate, length somewhat variable in preserved specimens, roughly one-third of ctenidium in length, nearly symmetrical, with lamellae along ctenidial axis slightly wider. Lamellae not numerous, rather narrow, roughly 35 in total. Pallial oviduct and rectum bordering mantle cavity at right. Anus opening well back from mantle edge, near anterior third of mantle cavity. Female gonopore opening short distance in front of anus. Hypobranchial gland (Fig. 6: hg) forming thick, elongate pad with sharp, well defined borders in mantle roof and partially overlying rectum, supported by white, calcium-bearing connective tissue; extending anterior to anus and female reproductive pore.

Radula (Fig. 7C): Radula with three teeth per row. Rachidian with arched basal plate and six main cusps on posterior margin. Lateral cusps slightly shorter than central cusp. Occasionally, very short denticles present on one or both sides, external to lateralmost cusps. Lateral teeth tricuspid, with central cusp much narrower and situated much closer to inner cusp.

**Figure 8:**
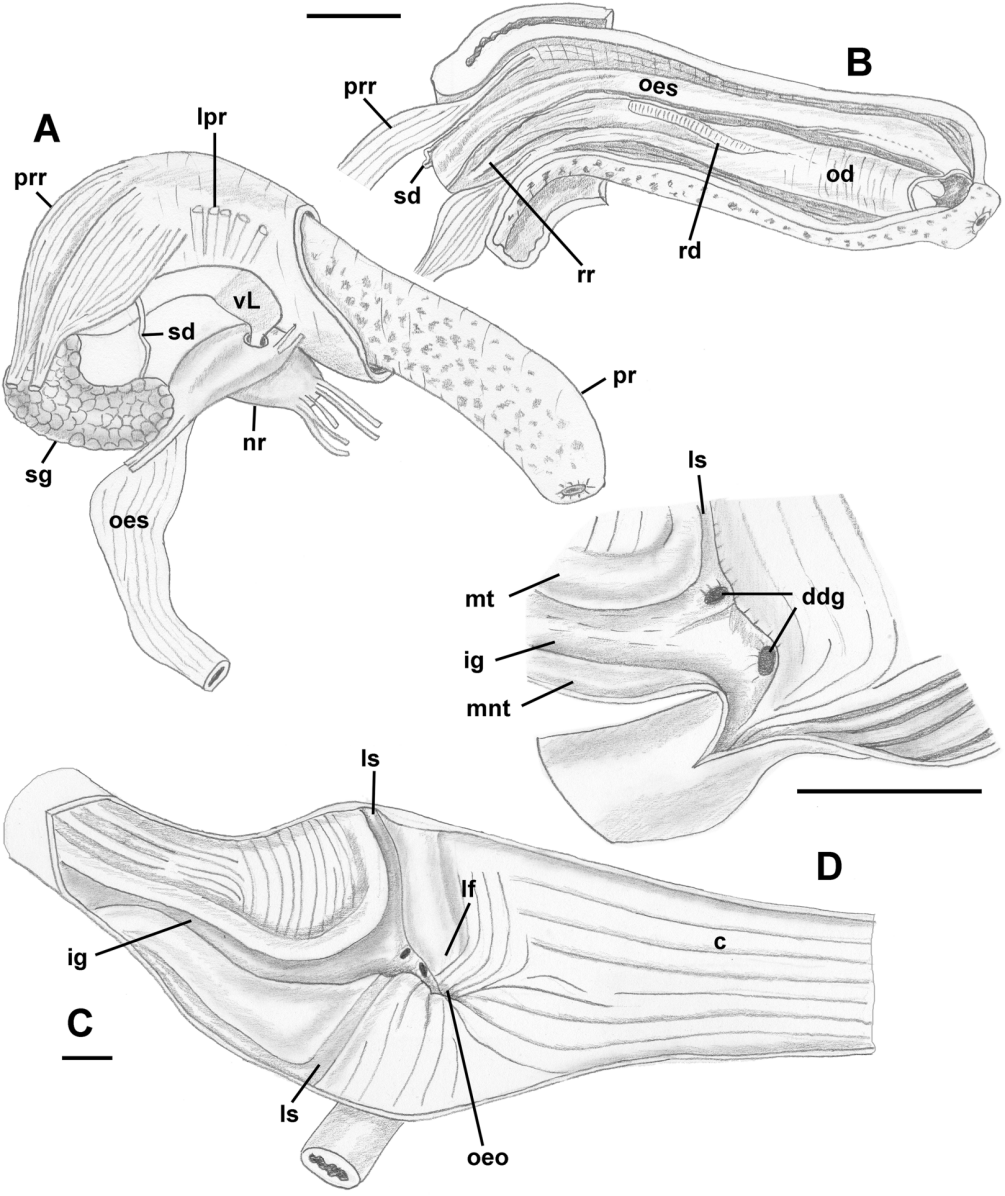
Anatomy of the digestive system of *Anentome* sp. A. (A, B) Foregut. (A) Right view of the foregut, rhynchodaeum partially removed to expose the proboscis. (B) Protracted proboscis opened along right-dorsal line. (C, D) Stomach. (C) Stomach opened along dorsal line and outer wall reflected. Only short portion of posterior mixing area is shown. (D) Enlarged area of opening of the posterior esophagus into stomach. Outer stomach wall removed, esophagus opened along mid-line. c, caecum; ddg, ducts of digestive gland; ig, intestinal groove; lf, longitudinal fold on the inner stomach wall; lpr, lateral proboscis retractors; ls, lateral sulcus; mnt, minor typhlosole; mt, major typhlosole; nr, nerve ring; od, odontophore; oeo, esophageal opening into gastric chamber; oes, esophagus; pr, proboscis; prr, proboscis retractors; rd, radula; rr, radular retractor; sd, salivary duct; sg, fused salivary glands; vL, valve of Leiblein. Scale bars: 1 mm.

Foregut (Figs. 8A and 8B): Proboscis in contracted state not long, slightly shorter than aperture, with smooth walls, pigmented with irregular black blotches. Mouth forming triangular slit. Length/diameter ca. 4.5 in retracted proboscis and 6.5 in protracted one. Powerful paired proboscis retractors attached to posterior outer rhynchodaeum (Figs. 8A and 8B: prr) in retracted proboscis and posteriormost proboscis walls in protracted proboscis and fuse with columellar muscle at posterior limit of hemocoel. Seven or eight smaller retractors attached to lateral walls of rhynchodaeum (Fig. 8A: lpr) and to lateral walls of hemocoel. Esophagus forming short loop upon leaving proboscis before passing through large nerve ring. Valve of Leiblein situated immediately anterior to nerve ring, poorly defined and very slightly broader than anterior esophagus, lacking ciliary cone. Esophagus expanding slightly in diameter after passing through nerve ring, paralleled by anterior aorta of slightly lesser diameter. Gland of Leiblein absent. Salivary glands acinous, fused, forming half-ring embracing posterior rhynchodaeum latero-ventrally. Salivary ducts free along anterior esophagus, entering its walls at half proboscis length, then continuing in walls of esophagus beneath lateral folds. Odontophore rather long, spanning roughly two-thirds of protracted proboscis in length, nearly equal in length to retracted proboscis. Radular sac equally long as odontophore. Odontophoral retractors fusing with proboscis walls.

Midgut (Figs. 8C and 8D): Stomach small, partially covered by digestive glands. Posterior mixing area very long, forming “caecum” spanning nearly three-quarters of whorl, equally narrow along its length (Fig. 8C). Caecum oval in transverse section, dorsoventrally compressed, lined with nine prominent, tall, longitudinal folds, several continuous with folds of posterior esophagus (Fig. 8C: oeo). Epithelial cells of folds mostly ciliated, with sporadic glandular, non-ciliated cells. Gastric chamber comparatively short. Small, paired, closely spaced, oval openings of digestive gland ducts adjacent to opening of esophagus. Each duct opening situated in rather deep depression. Longitudinal fold (Fig. 8C: lf) rather short, oblique, bordering posterior part of distinct, albeit narrow, lateral sulcus (Fig. 8C: ls). Lateral sulcus present on outer stomach wall, although shallower. Typhlosoles well-developed, bordering deep intestinal groove. Major typhlosole narrower and more prominent, while minor typhlosole broader but short, becoming obsolete at level of posterior kidney border. Inner wall of dorsal channel of style sac lined with transverse folds, replaced with inconspicuous longitudinal folds anteriorly.

Style sac region crossing below floor of posterior kidney, from posterior left to anterior right side, ventrally embedded in digestive gland. At base of mantle cavity, major typhlosole becoming inconspicuous, intestine turning and emerging into mantle roof. Intestine expanding somewhat into broad, flattened rectum, surrounded by voluminous rectal sinus. Rectum terminating anteriorly in small, simple, non-papillate anus.

**Figure 9:**
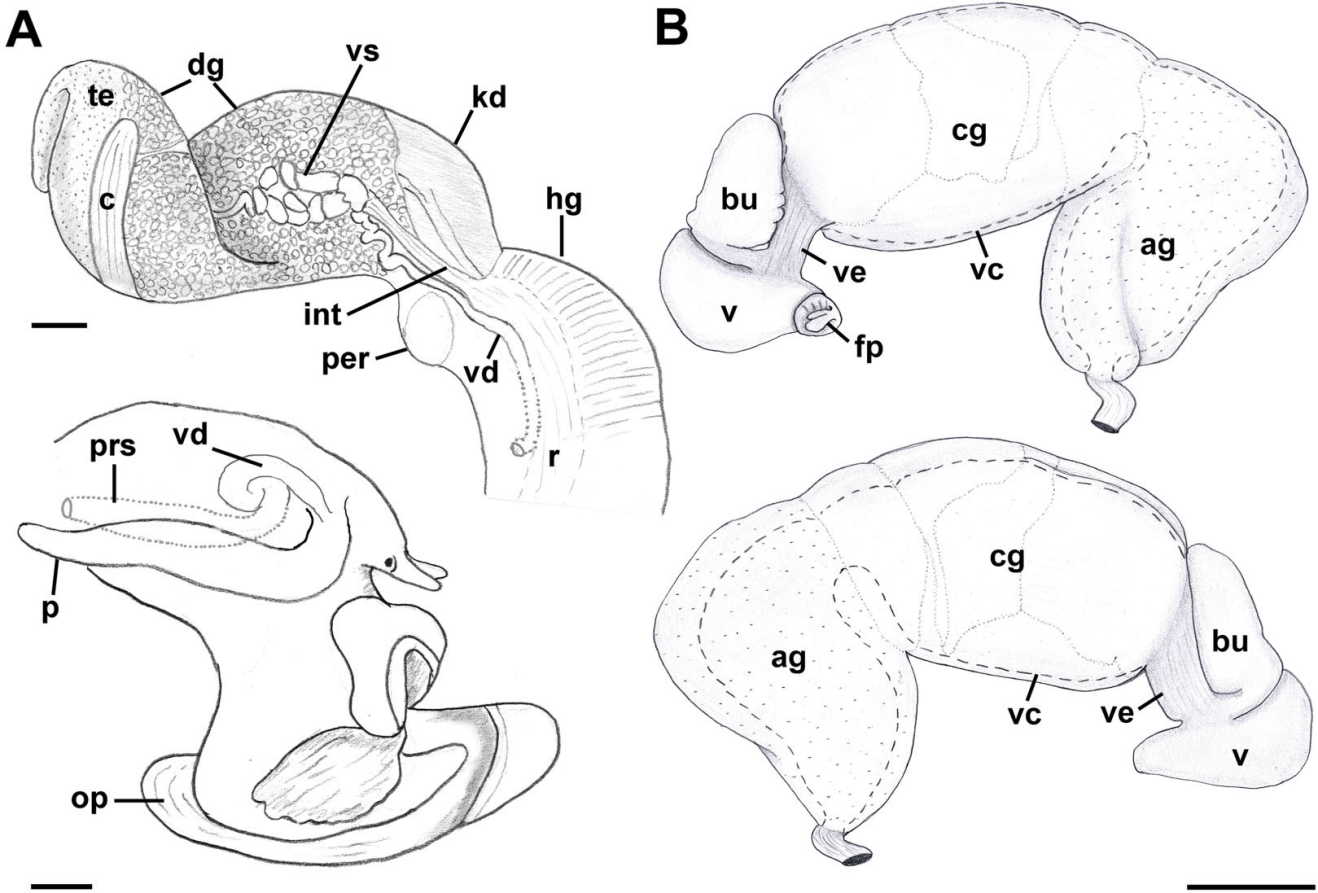
Anatomy of the reproductive system of *Anentome* sp. A. (A) Male reproductive anatomy. Visceral mass and posterior part of the mantle, uncoiled, above; right view of the head-foot with prostate and anterior muscular seminal duct seen by transparency, below. (B) Female reproductive anatomy. Left lateral view, above, anterior is at left; right lateral view, below, anterior is at right. Hashed line indicates boundaries of lumen. ag, albumen gland; bu, copulatory bursa; c, caecum; cg, capsule gland; dg, digestive gland; fp, female pore; hg, hypobranchial gland; int, intestine; kd, kidney; op, operculum; p, penis; per, pericardium; prs, prostate; r, rectum; te, testis; v, vagina; vc, ventral channel; vd, pallial vas deferens; ve, vestibule; vs, seminal vesicle. Scale bars: 1 mm.

**Figure 10:**
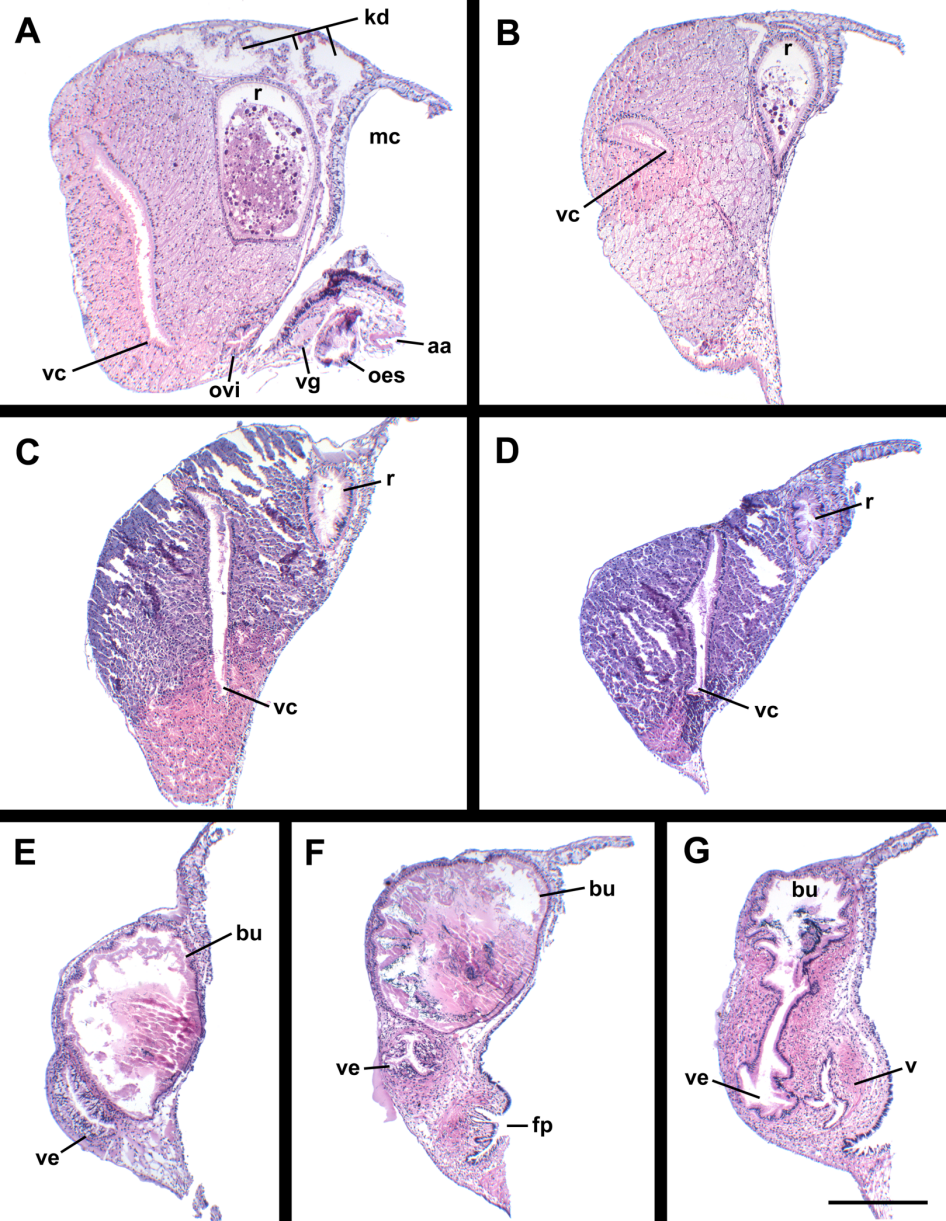
Histology of the pallial oviduct of *Anentome* sp. A. (A) Posterior albumen gland. (B) Anterior albumen gland. (C) Posterior capsule gland. (D) Anterior capsule gland. (E) Posterior copulatory bursa and non-glandular extension of ventral channel. (F) Middle copulatory bursa, vestibule and vaginal opening. (G) Anterior copulatory bursa and vestibule near entrance to ventral channel extension. aa, anterior aorta; bu, copulatory bursa; fp, female pore; kd, kidney; mc, mantle cavity; oes, esophagus; ovi, renal oviduct; r, rectum; v, vagina; vc, ventral channel; ve, vestibule; vg, visceral ganglion. Scale bar: 500 μm.

Reproductive anatomy (Figs. 9 and 10): Gonad overlying digestive gland at right in apical whorls, anteriorly bordering caecum at right to level of esophageal opening, extending to tip of apical whorls in mature specimens. Vas deferens emerging from testes ventrally, continuing anteriorly along ventral aspect of visceral whorls, forming thickened, coiled seminal vesicle (Fig. 9A: vs) behind base of mantle cavity on ventral surface of visceral whorl, dorsally embedded in digestive gland below and slightly behind kidney (Fig. 9A). Vas deferens straightening and narrowing before penetrating base of mantle cavity at right. In mantle roof, vas deferens continuing anteriorly as thickened glandular prostate (Fig. 9A: prs). Prostate long, slender, thin-walled, lying below rectum, crossing to neck at level of anus. Prostate narrowing anteriorly to pallial vas deferens with thick muscular walls. Pallial vas deferens completing two half loops within cephalic hemocoel, first looping through tight half turn posteriorly, then reversing course through broad half loop anteriorly, before continuing as ejaculatory duct down center of penis lying on right side of neck behind cephalic tentacle. Penis (Fig. 9A: p) long, slender, simple, without external glands or elaborations, tapering to simple, narrowly rounded tip with apical pore.

Oviduct emerging ventrally from ovary, continuing anteriorly along ventral aspect of visceral whorls. Gonopericardial duct lacking (Fig. 9B). Renal oviduct penetrating base of mantle cavity to enter pallial oviduct. Albumen gland (Figs. 9B and 10: ag) short, broad, with flattened lumen, comprising roughly one-third of glandular pallial oviduct. Albumen gland lumen narrowing anteriorly at junction with more distal capsule gland. Capsule gland (Figs. 9B and 10: cg) roughly twice albumen gland in length, also with flattened lumen. Capsule gland regionated into at least five externally and histologically differentiable sets of glands (Figs. 9B and 10). Precise pattern of folds somewhat variable between individuals, but symmetrical on right and left sides of lumen. Capsule gland continuous anteriorly with narrow, flattened, longitudinally ridged, non-glandular continuation of ventral channel, or vestibule. Vestibule (Figs. 9B and 10E–10G: ve) opening dorsally at anterior end to cylindrical, posteriorly directed copulatory bursa (Figs. 9B and 10E–10G: bu) containing unorientated sperm. Just in front of opening to bursa, vestibule expanding dorsally into small, muscular, bulbous vagina (Figs. 9B and 10G: v) terminating ventrally in elongate female pore. Right wall of vagina conspicuously thicker and deeply longitudinally ridged, with bifid fold extending into female pore. Entrance to vestibule just above female pore, between branches of bifid fold.

Remarks: We here follow the terminology of Fretter (1941) who termed the non-glandular continuation of the ventral channel anterior to the capsule gland, the vestibule, and the region between the female pore and the vestibule, the vagina. Kantor (2003) studied the stomach of *Anentome* sp. A (as “*Clea helenae*”), while Coelho, Dinis & Reis (2013) described the life history traits of “*Clea helena*” obtained through the aquarium trade, which is likely also conspecific with *Anentome* sp. A, judging from the rather poor figure of a single adult shell in abapertural view. In *Anentome* sp. B (MNHN IM-2013-52176), the odontophore is roughly one-half the length of the retracted proboscis.

*Nassodonta dorri* (Wattebled, 1886)

Material examined: Vietnam: Bình Thuận Province, Tuy Phong, Phan Ri River, Sông Lũy, 11°10.57′N, 108°33.70′E (MNHN uncataloged).

**Figure 11:**
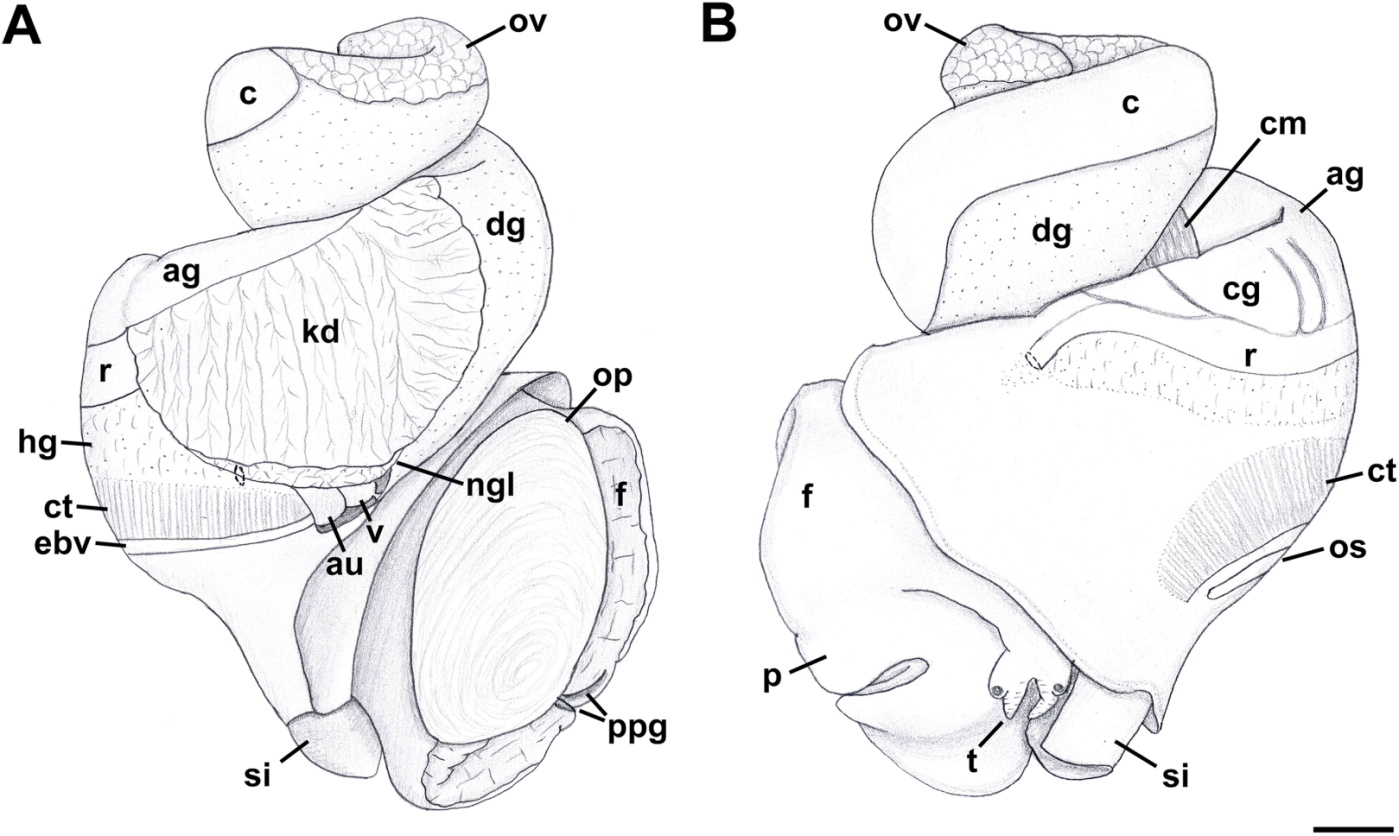
External anatomy of *Nassodonta dorri*. (A) Apertural view. Hashed line at anterior end of nephridial gland indicates nephropore, seen by transparency. (B) Abapertural view. ag, albumen gland; au, auricle; c, caecum; cg, capsule gland; cm, columellar muscle; ct, ctenidium; dg, digestive gland; ebv, efferent branchial vein; f, foot; hg, hypobranchial gland; kd, kidney; ngl, nephridial gland; op, operculum; os, osphradium; ov, ovary; p, propodium; ppg, propodial pedal gland; r, rectum; si, siphon; t, cephalic tentacle; v, ventricle. Scale bar: 1 mm.

External anatomy (Fig. 11): Mature animal comprising 2.25 whorls in preserved specimens. Head small, broad, with very short, thick cephalic tentacles (Fig. 11: t), with eyes slightly elevated on prominent ocular peduncles at tentacle outer bases. Foot broad, fleshy, overlapping sides of operculum in preserved specimens. Propodium narrow with posterior extent marked by indistinct notch, poorly demarcated from mesopodium. Shallow propodial pedal gland (Fig. 11: ppg) along anterior edge, with two histologically distinct subepithelial gland cells. Ovipositor forming deep, simple pore surrounded by weakly developed subepithelial glands. Metapodium lacking posterior tentacles. Operculum (Fig. 11: op) thin, elongate, oval with basal nucleus. Mantle cavity short, less than one-half whorl in length, posteriorly bounded by reno-pericardial complex. Mantle cavity slightly asymmetrical, slightly deeper at left side in front of pericardium. Columellar muscle long, extending just past posterior end of kidney. Ctenidium (Fig. 11: ct) extending along left side of mantle cavity from near mantle edge to pericardium behind left base of mantle cavity. Short siphon (Fig. 11: si) emerging from behind mantle edge at left anterior mantle cavity in front of gill, to left of head. Long osphradium lying to left of central two-thirds of ctenidium. Osphradium bipectinate, asymmetrical, with leaflets along ctenidial axis much larger than those along mantle floor. Osphradium separated from efferent branchial vein by deep cleft. Pallial oviduct and rectum bordering mantle cavity at right. Papillate anus opening well back from mantle edge, near anterior third of mantle cavity. Female gonopore opening just behind anus. Hypobranchial gland (Fig. 11: hg) forming elongate, thick pad with sharp borders in mantle roof and partially overlying rectum, thinning and extending short distance anterior to anus and female reproductive pore.

Radula: (after Kantor & Kilburn, 2001) Radula comprising roughly 75 rows. Lateral teeth with four to six cusps, with number of cusps varying even on adjacent rows. Outermost cusps ∼2.5 longer than innermost cusps. Intermediate cusps sharply pointed or bifurcating at their tips. Innermost cusp with seven to eight denticles on inner, lateral side. Rachidian with 11–12 cusps, central cusp serrated, with number of cusps varying even on adjacent rows. Basal plate evenly and deeply notched along anterior edge.

**Figure 12:**
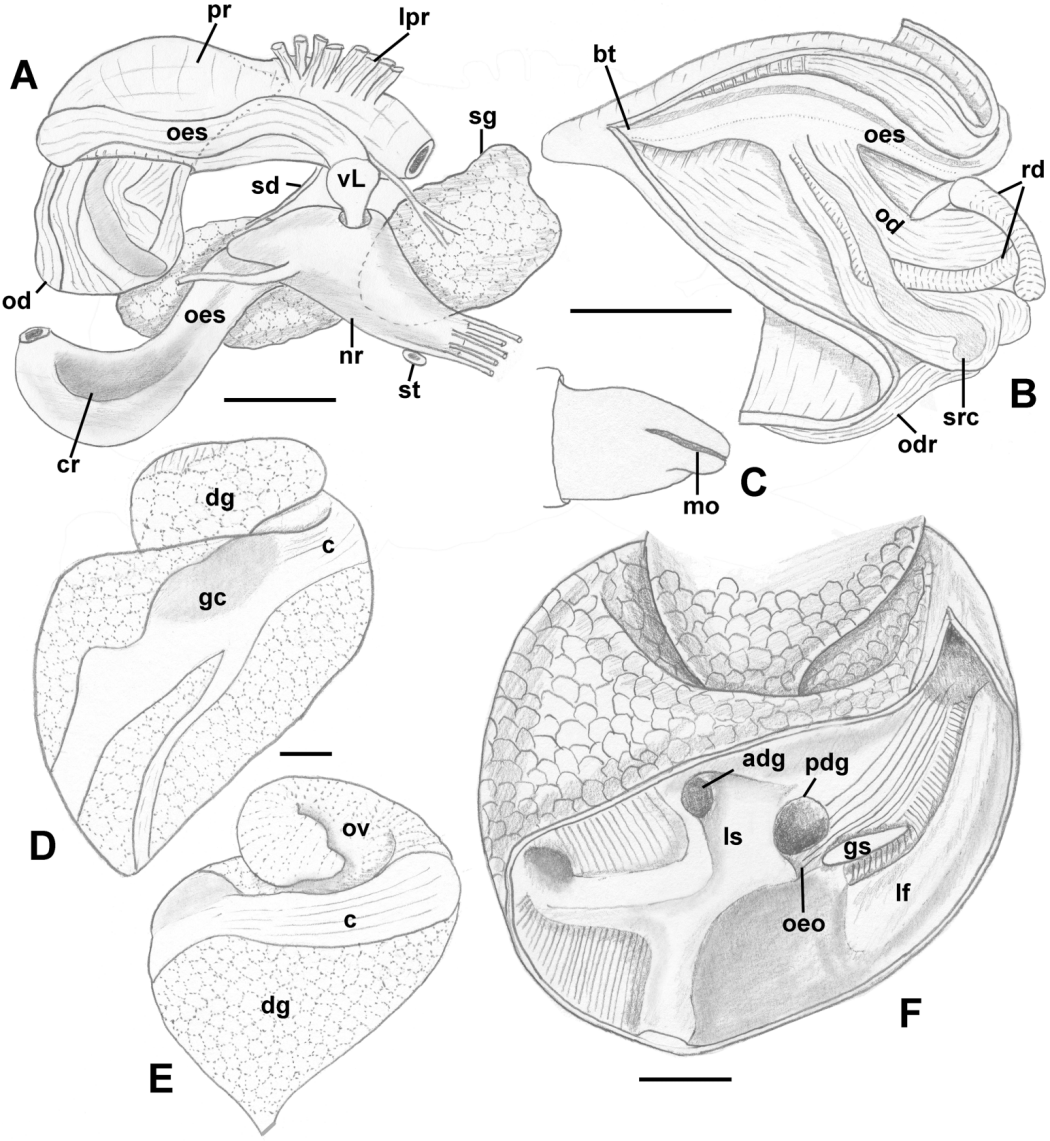
Anatomy of the digestive system of *Nassodonta dorri*. (A–C) Foregut. (A) Right view of the foregut, right salivary gland displaced to show the nerve ring, dashed line indicates proboscis retracted within rhynchodaeum. (B) Proboscis opened along left-dorsal line. (C) Proboscis tip, ventral view. (D–F) Midgut. (D–E) Views of visceral mass showing layout of posterior digestive system. (F) Gastric chamber opened along right side. adg, anterior duct of digestive gland; bt, buccal tube; c, caecum; cr, crop; dg, digestive gland; gc, gastric chamber of stomach; gs, gastric shield; lf, longitudinal fold on outer stomach wall; lpr, lateral proboscis retractors; ls, lateral sulcus; mo, mouth; nr, nerve ring; od, odontophore; odr, odontophoral retractor; oeo, esophageal opening into gastric chamber; oes, esophagus; ov, ovary; pdg, posterior duct of digestive gland; pr, proboscis; rd, radula; sd, salivary duct; sg, salivary gland; st, statocyst; vL, valve of Leiblein. Scale bars: 1 mm.

Foregut (Figs. 12A–12C): Proboscis very short and coniform, deeply retracted into rhynchodaeum, spanning only about half of rhynchodaeum length. Mouth forming narrow ventral slit at anterior tip of proboscis (Fig. 12C). In retracted proboscis, odontophore and radular sac significantly protruding from rear of proboscis (Fig. 12A: od). Buccal tube rather long, spanning nearly half of proboscis length (Fig. 12B: bt). Proboscis retractors arranged in bundles, attached to antero-lateral side of rhynchodaeum (Fig. 12A: lpr). Esophagus forming long loop, as long as rhynchodaeum, upon emerging from proboscis, before passing through large nerve ring. Valve of Leiblein pyriform, well defined, situated immediately anterior to nerve ring. Esophagus significantly expanding in diameter after passing through nerve ring, forming thin walled, spoon-shaped “crop,” embracing posterior part of protruded odontophore (Fig. 12A: cr). Gland of Leiblein absent. Salivary glands acinous, separate, large. Right salivary gland nearly completely covering nerve ring laterally (displaced in Fig. 12A to show nerve ring and valve of Leiblein). Salivary ducts thick, paralleling anterior esophagus. Odontophore large, muscular, nearly as long as proboscis. Large unpaired odontophoral retractor attached to ventral side of rhynchodaeum (Fig. 12B: odr). Subradular cartilages fused anteriorly along third of odontophore length. Radular sac equally long as odontophore.

Midgut (Figs. 12D–12F): Stomach very long, spanning approximately one whorl from posterior kidney border, roughly three-quarters of length comprising very long caecum, situated along right aspect of visceral whorls, parallel to longitudinal axis (Figs. 12D and 12E. Gastric chamber and style sac situated obliquely to longitudinal axis, across visceral whorls. Relief of stomach epithelium very low. Very broad, oval posterior opening of digestive gland duct (Fig. 12F: pdg) adjacent to opening of esophagus (Fig. 12F: oeo). Anterior duct smaller (Fig. 12F: adg), situated in depression in lateral sulcus. Longitudinal folds of posterior esophagus continuous into caecum, with folds also orientated parallel to main stomach axis. Longitudinal fold separating ducts and esophageal opening from dorsal chamber of stomach broad, poorly pronounced. At esophageal opening in ventro-dorsal wall, distinct cuticularized gastric shield present (Fig. 12F: gs). Broad but low longitudinal fold on outer wall continues from esophageal opening into caecum, bordered by transverse folds in narrow zone immediately adjacent to fold. Caecum otherwise bearing very low, narrow, inconspicuous longitudinal folds. Typhlosoles less pronounced than intestinal groove. Inner wall of dorsal channel of style sac lined with weak transverse folds. Stomach integuments in area of style sac pigmented.

**Figure 13:**
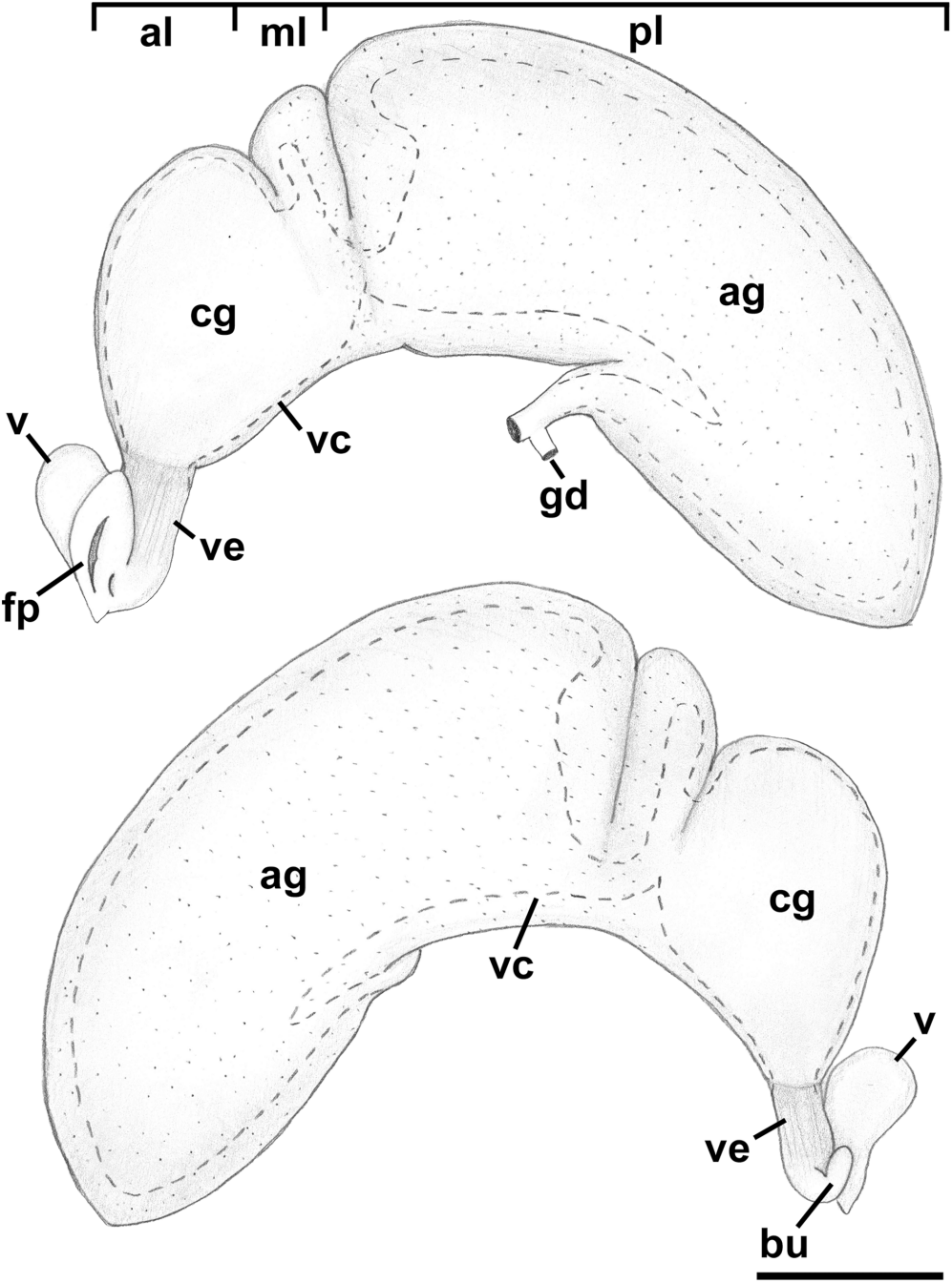
Female reproductive anatomy of *Nassodonta dorri*. Left lateral view, above, anterior is at left; right lateral view, below, anterior is at right. Hashed line indicates boundaries of lumen. Note small, separate opening of vestibule to mantle cavity below anterior end of female pore. ag, albumen gland; al, anterior lobe; bu, copulatory bursa; cg, capsule gland; fp, female pore; gd, gonopericardial duct; ml, middle lobe; pl, posterior lobe; v, vagina; vc, ventral channel; ve, vestibule. Scale bar: 1 mm.

**Figure 14:**
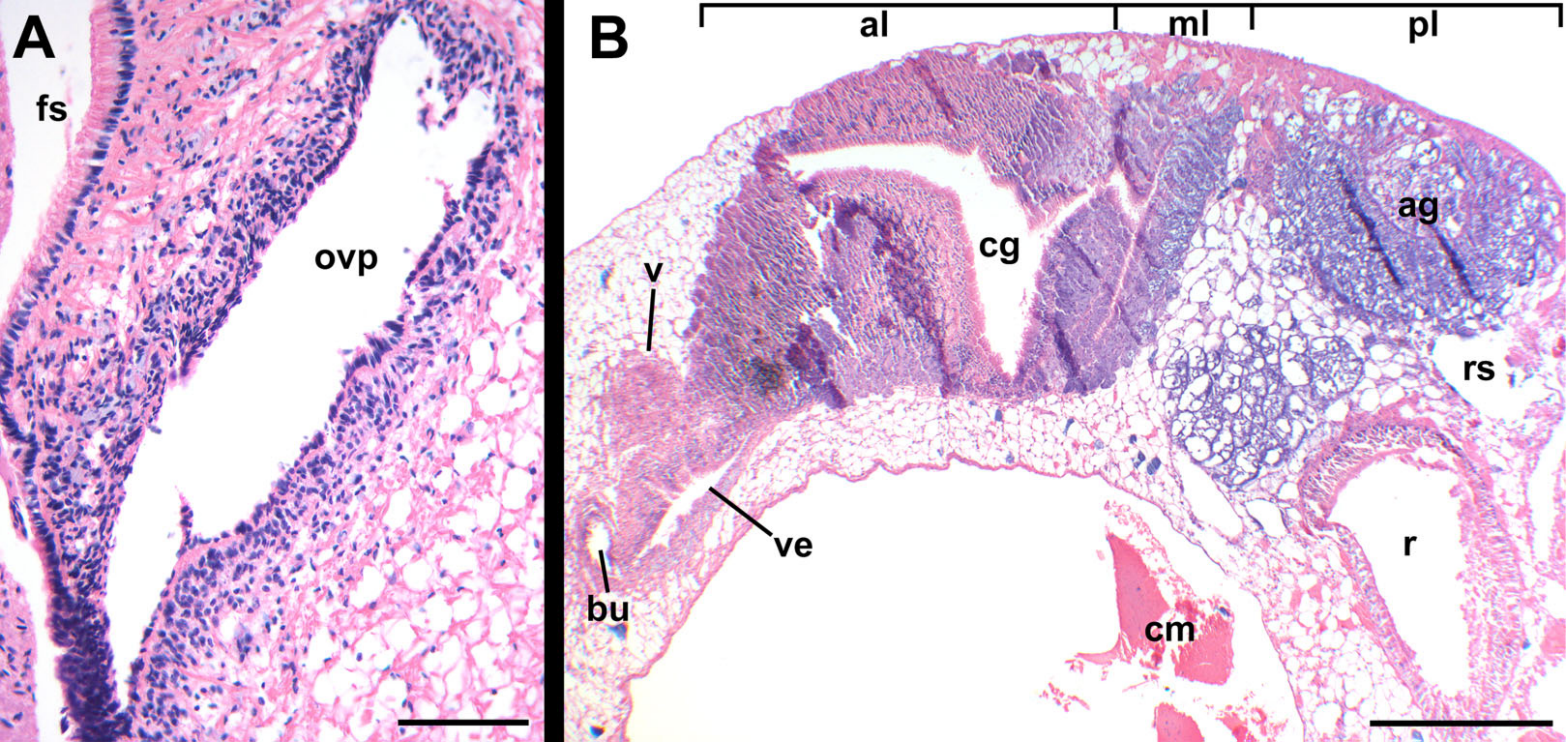
Histology of female reproductive anatomy of *Nassodonta dorri*. (A) Longitudinal section of ovipositor. (B) Longitudinal section of glandular pallial oviduct. ag, albumen gland; al, anterior lobe; bu, copulatory bursa; cg, capsule gland; cm, columellar muscle; fs, foot sole; ml, middle lobe; ovp, ovipositor; pl, posterior lobe; r, rectum; rs, rectal sinus; v, vagina; ve, vestibule. Scale bars: (A) 100 μm, (B) 500 μm.

Reproductive anatomy (Figs. 13 and 14): Gonad overlying digestive gland at right in apical whorls, posteriorly bordering caecum, extending to tip of apical whorl in mature specimens. Vas deferens very thin, emerging from testis ventrally, continuing anteriorly along ventral aspect of visceral whorls. Seminal vesicle not defined. Vas deferens broadening very slightly before penetrating base of mantle cavity at right, continuing anteriorly below rectum, crossing to base of poorly glandular prostate near posterior end of mantle cavity. Prostate medium short, with thin walls, narrowing anteriorly to pallial vas deferens. Pallial vas deferens coiling slightly before continuing as ejaculatory duct down center of penis lying on right side of neck behind cephalic tentacle. Penis long, slender, simple, without external glands or elaborations, tapering to very short and narrow papilla with apical pore.

Oviduct emerging ventrally from ovary, continuing anteriorly along ventral aspect of visceral whorls. Near base of mantle cavity, renal oviduct turning posteriorly. Gonopericardial duct present (Fig. 13: gd). Renal oviduct entering pallial oviduct well behind base of mantle cavity, along right side of kidney. Glandular pallial oviduct externally divisible into three distinct lobes (Figs. 13 and 14B). Posterior lobe (Figs. 13 and 14B: pl) long, weakly u-shaped, comprising roughly two-thirds of glandular pallial oviduct, with broad flattened lumen bordered by albumen glands (Figs. 13 and 14B: ag). Lumen turning slightly to right at anterior end of lobe and narrowing, before turning sharply left to enter small, distinct central lobe (Figs. 13 and 14B: ml). Within central lobe, lumen of oviduct oriented transversely across longitudinal axis, bordered posteriorly by albumen gland and anteriorly by capsule gland. Lumen turning sharply again at left side of oviduct, to continue anteriorly in anterior lobe (Figs. 13 and 14B: al), bordered by distinctly regionated capsule glands. Lumen in anterior lobe again oriented parallel to longitudinal axis. Glands of capsule gland (Figs. 13 and 14B: cg) darkly pigmented with dispersed black granules at anterior end. Capsule gland continuous anteriorly with short, narrow, flattened, longitudinally ridged, non-glandular vestibule. Vestibule (Figs. 13 and 14B: ve) opening dorsally near anterior end to vestigial copulatory bursa, empty in histological sections. Vestibule opening to mantle cavity via small, separate, slit-like pore below anterior end of large separate entrance to rounded, muscular vagina (Figs. 13 and 14B: v).

Remarks: Several specimens used in the anatomical investigations of this species, including those used in midgut dissections and for histological sectioning, had been previously dried and rehydrated. This has introduced some artefacts, and may have influenced some of the observations, including the low relief of the stomach epithelium and the size of the openings of the digestive gland ducts, the posterior of which was observed to be significantly smaller in a second specimen. In addition, only a single male (SL 12.4 mm) was available for study, and while being adult, was obviously between reproductive seasons. *Nassodonta* is a genus with only two species currently recognized (*Nassodonta dorri*, *Nassodonta insignis* Adams, 1867), traditionally placed in the Nassariidae (Smith, 1895b). *Nassodonta* species live in turbid brackish waters from India to Vietnam, with an unconfirmed report from China (Smith, 1895b), and are capable of withstanding a wide range of salinities. Kantor & Kilburn (2001) reported on the rediscovery of *Nassodonta dorri*, described the shell, radula and provided some preliminary anatomical observations based on a single, poorly preserved female, and discussed the family placement of the genus. Simone (2007) provided a description of the external anatomy, proboscis musculature and radula, based on “semi-mummified” specimens.

### Systematics

Within *Clea*, 15 species are currently recognized as valid, with an additional nine nominal species and six varieties that are currently recognized as synonyms of *Clea helena*, *Clea jullieni* or *Clea nigricans* (MolluscaBase, 2017).

*Clea* was described by H. & A. Adams, 1855; the type species is *Clea nigricans* A. Adams, 1855, by monotypy. As currently recognized, two available genus-group names have been synonymized with *Clea*: *Quadrasia* Crosse, 1886, type species *Quadrasia hidalgoi* Crosse, 1886 by original designation; and *Anentome* Cossmann, 1901, a replacement name for *Canidia* H. Adams, 1862 (non-Thomson, 1857 [Coleoptera], nec Holmgren, 1858 [Hymenoptera]). Adams (1862), when establishing *Canidia*, did not explicitly designate a type species, but in stating that, “*Melanopsis helena* from Java, is a second species of *Canidia*” (1862: 384), it seems that his intention may have been to designate his new species *Canidia fusca* H. Adams, 1862, from Cambodia, as type. Regardless, Cossmann (1901) recognized the homonymy of Adams’ genus-group name, and that a valid type designation had not been made, and established *Anentome* as a replacement name, proposing *Canidia jullieni* Deshayes, 1876 as type. However, because the latter was not one of the two originally included species, Cossmann’s type designation is invalid (Article 67.2; ICZN, 1999). Consequently, *Canidia*, and hence *Anentome*, remain without a validly fixed type species. Without any basis for grounding the concept, the name *Anentome* has been applied inconsistently, and variably at the rank of genus or subgenus (Brandt, 1974; Mienis, 2011; Newel & Bourne, 2013; Ng et al., 2016a), resulting in taxonomic instability.

It is now clear, based on the evidence presented herein, that the current concept of “*Clea helena*” comprises a complex of at least four molecularly and morphologically distinct (Figs. 3A and 4) taxonomical species. “*Clea helena*” differs from *Clea nigricans*, the type species of *Clea*, in discrete differences in shell, operculum and radula morphology (see below). Consequently, we here support the recognition of two distinct genera: the genus *Clea* for *Clea nigricans* and its allies, and the genus *Anentome*, for which we designate *Melania helena* von dem Busch, 1847 as type, and its allies. Given the isolated phylogenetic position of *Anentome* “*helena*” here and in the comprehensive analysis of Galindo et al. (2016), *Anentome* is here placed in the new subfamily Anentominae. *Nassodonta* was united as the sister group to *Anentome* in the five-gene molecular analysis here (Fig. 4), with which it shares several unique features of reproductive and foregut anatomy and which support its inclusion within the Anentominae. Although not included in the morphological and molecular analyses, *Clea* is retained here provisionally pending further review.

This name, Anentominae, was first used in the revised classification of Nassariidae constructed by Galindo et al. (2016), but no description or definition was provided (Article 13.1.1; ICZN, 1999) and it was not declared intentionally as new (Article 16.1; ICZN, 1999) and hence is not available from that publication.

Family Nassariidae Iredale, 1916 (1835)
Subfamily Anentominae subfam. nov.
Type genus: *Anentome* Cossmann, 1901

**Figure 15:**
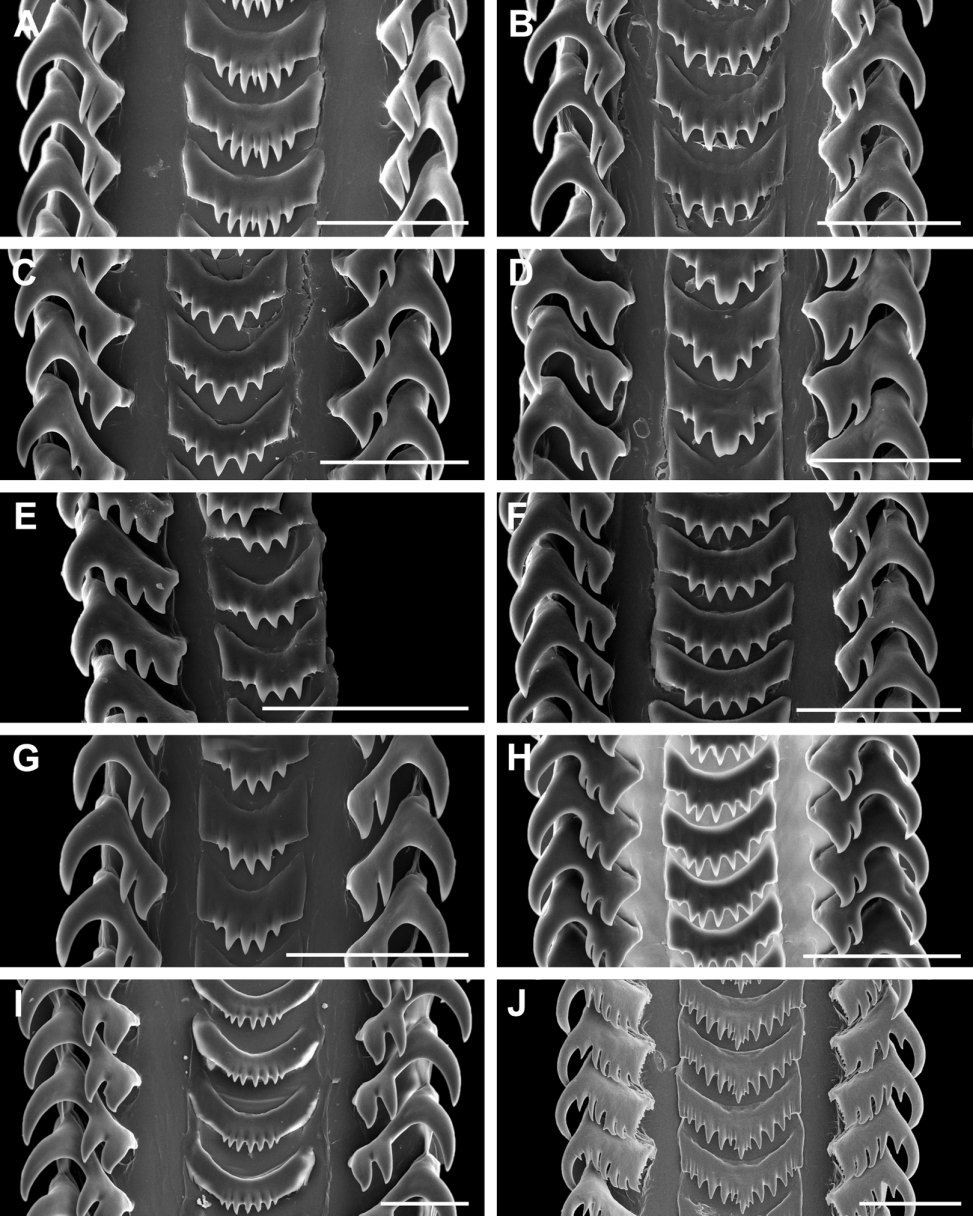
Comparative radula morphology of freshwater and estuarine nassariids. (A) *Anentome* sp. B, Thailand, MNHN IM-2013-52176. (B) *Anentome* sp. B, Thailand, MNHN IM-2013-52175. (C) *Anentome* sp. C, Malaysia, MNHN IM-2013-52180. (D) *Anentome* sp. C, Malaysia, MNHN IM-2013-52181. (E) *Anentome* sp. C, Malaysia, MNHN IM-2013-52179. (F) *Anentome* sp. D, Vietnam, MNHN IM-2009-29661. (G) *Anentome* sp. C, Sumatra, MNHN, uncataloged. (H) *Anentome* “*helena*”, Java, MNHN, uncataloged. (I) *Clea nigricans*, Borneo, MNHN, uncataloged. (J) *Nassodonta dorri*, Vietnam, MNHN, uncataloged.

Diagnosis: Shell fusiform, rather thin to solid. Siphonal canal short but distinct, moderately narrow to rather broad. Anterior sinus in outer lip well developed to obsolete. Basal sulcus occasionally present (*Anentome*, *Nassodonta*). Shell smooth (*Clea*) to transversely plicate (*Anentome*, *Nassodonta*), often with spiral threads or cords; spiral ornament may be limited to subsutural ramp or base. Periostracum straw, olive to brown-black in color. Rachidian strongly arched, with straight (*Anentome*, *Nassodonta*) or rounded (*Clea*) lateral edges, ∼3–10 (*Anentome*, *Clea*; Figs. 7C and 15A–15I) to ∼11–12 (*Nassodonta*; Fig. 15J) small, sharp denticles, central cusp may be serrated (*Nassodonta*). Lateral teeth with 3–4 (*Anentome*, *Clea*) to 4–6 cusps (*Nassodonta*), innermost cusp may be serrated along inner, lateral edge (*Nassodonta*). Gland of Leiblein absent. Valve of Leiblein present, small. Stomach with extremely long and narrow caecum, ducts of digestive gland closely spaced. Gastric shield may be present (*Nassodonta*). Metapodial tentacles lacking. Anterior pallial oviduct with long, longitudinally grooved vestibule, and with both a thick, muscular vagina and copulatory bursa.

Remarks: We here segregate *Anentome* in a separate subfamily given its isolated phylogenetic placement in the comprehensive phylogeny of the family (Galindo et al., 2016) and in the five-gene phylogeny based on a restricted taxonomic sample here (Fig. 4), and given several unique features of the anatomy when compared to other nassariids. The pallial oviduct uniquely bears a large anterior bursa connected to a rather long non-glandular vestibule. The oviduct of *Nassodonta* is remarkably similar in organization, but the bursa is vestigial. Given that *Nassodonta* is here placed as the sister group to *Anentome* based on molecular data, albeit without significant support, we here also place *Nassodonta* in the Anentominae. This arrangement is not without precedent; the placement of *Clea* and *Anentome* within the Nassariidae was supported by Fischer (1884; as *Canidia*) and followed by Cossmann (1901), based on features of the shell and the appearance of the animal, and was suggested on the basis of stomach anatomy by Kantor (2003; for “*Clea helenae*”). The proposed arrangement of the genera recalls that of Fischer (1884) who recognized three sections within *Canidia*: *Canidia* s.s., *Clea* and *Nassodonta*. Indeed, in addition to 11 of the nominal species currently classified in *Clea*, *Nassodonta dorri* was originally described in the genus *Canidia*. The distinctive basal sulcus of *Nassodonta* (Kantor & Kilburn, 2001) corresponds to a well-developed anterior sinus in the outer lip, the latter a feature shared with some members of *Anentome*, particularly *Anentome jullieni*.

Genus *Anentome* Cossmann, 1901
*Canidia* H. Adams, 1862 (invalid: non *Canidia* J. Thomson, 1857 [Coleoptera], nec Holmgren, 1858 [Hymenoptera]; *Anentome* is a replacement name).
Type species: *Melania helena* von dem Busch (in Philippi), 1847, here designated.
Type material: Lectotype ÜMB TK 279/1 (Knipper, 1958); paralectotypes MNHN IM-2000-27679 (5 spms).
Type locality: “Java.”

Revised Diagnosis: Shell fusiform, ovate-conoidal, subglobose or turreted, rather thin to somewhat solid. Apex typically eroded, whorls flattened or weakly to moderately convex, sutures moderately to deeply impressed. Ornament often of transverse plicae; spiral ornament of few, thick, elevated spiral ridges, to variable number of fine spiral threads to thicker cords, sometimes with tubercles or spines. A granulose texture may be produced by the intersection of spiral and transverse elements. Columella truncate, nearly straight to moderately curved, with basal notch weak to obsolete. Siphonal canal short but distinct, rather broad. Aperture elongate-ovate to angular; outer lip smooth, sinuous, with variably developed anterior sinus. Periostracum straw, olive, to chestnut or reddish brown in color; shell whitish to yellowish or greenish, with variable number of light to dark spiral bands typically present, often one to three, often visible within aperture. Operculum concave, with basal nucleus slightly turned to left, and thickened, elevated process behind. Rachidian with strongly arched basal plate, straight lateral edges, serrated cutting edge bearing ∼3–7 small, sharp denticles along central portion of tooth. Lateral teeth typically tricuspid, but fourth cusp may be present. Metapodial tentacles lacking.

Included species: *Anentome helena* (von dem Busch, 1847), *Anentome bizonata* (Deshayes, 1876) comb. nov., *Anentome costulata* Schepman, 1885 comb. nov., *Anentome cambojiensis* (Reeve, 1861) comb. nov., *Anentome fusca* (H. Adams, 1862) comb. nov., *Anentome jullieni* (Deshayes, 1876), *Anentome paviei* (Morlet, 1866) comb. nov., *Anentome scalarina* (Deshayes, 1876) comb. nov., *Anentome spinosa* (Temcharoen, 1971) comb. nov., *Anentome wykoffi* (Brandt, 1974) comb. nov.

Remarks: Although it seems that Adams’ (1862) intention was to designate his new species *Canidia fusca* H. Adams, 1862 as type of *Canidia*, he did not do so explicitly; furthermore, *Canidia fusca* was never illustrated and the type material appears to be lost. Two specimens from Cambodia from the Cuming collection in the NHMUK (Reg. no. 20001316) are identified on the label as “possible syntypes” but were concluded to not match the original description and to have no type status (Kantor & Kilburn, 2001). No other potential type material is presently known. Consequently, the identity of Adams’ intended type species is uncertain. For this reason, we here designate *Melania helena* von dem Busch, 1847 as type. The lectotype of *Melania helena* was designated by Knipper (1958; taf. 9, fig. 15) for a specimen from the von dem Busch collection in the Mollusca collection of the Überseemuseums Bremen, now stored in the Geosciences Collection of the University of Bremen (Lehmann, 2016). Five paralectotypes (Fig. 1) in the MNHN originated from the collection of Johan Christiaan Meder, part of which was purchased by the MNHN in 1842. It was Meder to whom von dem Busch (in Philippi, 1847) attributed the name *Melania helena* in the original description. The paralectotype lot was originally labelled “*Melanopsis*?” from Java without further geographic detail. The leftmost specimen in Fig. 1 is similar to the lectotype in shape, ornament and banding pattern, but is slightly smaller (16.0 vs. 18.3 mm) and with a damaged apex.

Genus *Clea* H. Adams & A. Adams, 1855.
Type species: *Clea nigricans* A. Adams, 1855, by monotypy.
Type material: four syntypes, NHMUK 20080063. Cuming collection.
Type locality: “the river in Sarawak Borneo.”
Synonym: *Quadrasia* Crosse, 1886.
Type species: *Quadrasia hidalgoi* Crosse, 1886, by original designation.
Type material: Lectotype MNHN IM-2000-30794 (Houbrick, 1986).
Type locality: “Ile Balabac, dans l’archipel des Philippines.”

Revised diagnosis: Shell ovate, solid, spire equal to or shorter than aperture in length. Apex blunt, typically eroded, whorls rather convex, sutures weakly to moderately impressed, may be slightly overhung by subsequent whorl. Shell mostly smooth, may be transversely striate; spiral ornament variable, may comprise variably developed sutural cord, one to two subsutural striae, to many fine, undulating spiral threads or more prominent spiral cords extending across shell surface. A finely granulose texture may be produced by the intersection of spiral and transverse elements of the ornament. Columella truncate, concave, with prominent basal notch. Siphonal canal short but distinct, anal canal elongate. Last whorl inflated, with elongate, fusiform aperture; outer lip smooth, simple, may be slightly sinuous. Periostracum light brown to dark olive or brown-black in color; shell whitish to purple or chocolate brown with up to three dark spiral bands. Operculum corneous, flat, elongate-ovate, with terminal to subterminal nucleus. Rachidian with strongly arched basal plate, rounded lateral edges, serrated cutting edge bearing ∼7–10 small, sharp pointed denticles along central portion of tooth. Lateral teeth tricuspid. Metapodial tentacles lacking.

Included species: *Clea nigricans* A. Adams, 1855, *Clea bangueyensis* E. A. Smith, 1895, *Clea bockii* Brot, 1881, *Clea funesta* H. Adams, 1862, *Clea hidalgoi* (Crosse, 1886).

Remarks: Based on features of the external anatomy and radula, Houbrick (1986) transferred *Quadrasia hidalgoi* from the Planaxidae to *Clea*, unaware that Smith (1895a) had already done so many years earlier. Given that the original description neither implies nor requires that there were syntypes, Houbrick’s (1986) inference of the status of the single specimen in the collections of the MNHN as the holotype, constitutes lectotype selection under Art. 74.6 of the *Code* (ICZN, 1999). As mentioned, we here provisionally retain *Clea* in the Anentominae pending more thorough morphological and molecular analysis.

Genus *Nassodonta* H. Adams, 1867.
Type species: *Nassodonta insignis* H. Adams, 1867, by monotypy.

Revised diagnosis: Oviduct with vestigial bursa; vestibule and vagina with separate openings to mantle cavity. Metapodial tentacles lacking. For features of shell and radula, see Kantor & Kilburn (2001).

Included species: *Nassodonta insignis* H. Adams, 1867, *Nassodonta dorri* (Wattebled, 1886), *Nassodonta annesleyi* (Benson, 1861) comb. nov.

Remarks: Kantor & Kilburn (2001) incorrectly stated paired metapodial tentacles to be present.

*Nassodonta annesleyi* is here transferred from *Clea* to *Nassodonta* after examining a syntype from the Benson collection in the University Museum of Zoology in Cambridge (R. C. Preece et al., 2017, unpublished data). *Clea annesleyi* was described by Benson (1861) from “Quilon” [now Kollam, India], from, “a tank between the sea and the canal which communicates with Cochin to the north of Quilon” (1861: 258). The shell has never been figured, the species rarely mentioned in the literature (Benson, 1862; Brot, 1862, 1868, 1876) and apparently maintained in *Clea* or *Canidia* on the merits of Benson’s description of the operculum (Brot, 1868). Tryon (1881) stated that he could not identify this unfigured species. The shell, with its distinctive basal sulcus, reveals it to be allied to *Nassodonta. Nassodonta gravelyi* (Preston, 1916), described from “Cochin” [Kochi, India] and formerly in the synonymy of *Nassodonta insignsis* (Cernohorsky, 1984), is here placed in the synonymy of *Nassodonta annesleyi*.

## Discussion

### Comparative anatomy of anentomines

Anentomines lack the paired metapodial tentacles typical of many nassariids, including Bulliinae, Dorsaninae and most Nassariinae and Photinae. The lack of metapodial tentacles is a feature that is not unique in the family, but is shared with the redefined Cylleninae, including *Tomlinia*, *Cyllene* and *Nassaria* (Galindo et al., 2016). The operculum of *Anentome* is concave with a basal nucleus that is slightly turned to left and bears a thickened, elevated process behind. The resulting curved, cup-like shape is quite different from the simple, flattened operculum with terminal to subterminal nucleus as found in *Clea*, *Nassodonta* and other nassariids (Brot, 1876, 1881; Smith, 1895a, 1895b; Houbrick, 1986; Kantor & Kilburn, 2001; Simone, 2007; Simone & Pastorino, 2014).

In contrast to the typically bicuspid lateral teeth of most nassariines (Cernohorsky, 1984), the lateral teeth of *Anentome* and *Clea* are tricuspid, occasionally with a transient fourth cusp that may appear on only a single side of the radula ribbon (e.g., *Anentome* “*helena*;” Fig. 15H), while those of *Nassodonta* are multicuspid, with four to six cusps. The development of multiple cusps on the lateral teeth is also seen in *Bullia, Buccinanops* and *Phrontis* (Bandel, 1984; Cernohorsky, 1984; Simone, 1996; Kantor & Kilburn, 2001), but the serrations found along the inner edge of the lateral teeth and the serrated central cusp of the rachidian in *Nassodonta* are unique in the family.

The foregut seems to be quite different between *Anentome* and *Nassodonta*, although they share the absence of the gland of Leiblein, which is typically present in nassariids (Graham, 1941; Strong, 2003). Kantor (2003) recognized the similarities in midgut morphology of *Nassaria* and *Anentome* (as *Clea*), now both recognized as members of a redefined Nassariidae (Galindo et al., 2016). The midgut of *Nassodonta* and *Anentome* is very similar in general shape, particularly in the presence of an extremely long posterior mixing area which forms a caecum, and shares with other nassariids the very short gastric chamber and the closely spaced ducts of the digestive gland. However, the cuticularized gastric shield, which was also observed in some Nassariidae (Brown, 1969; Strong, 2003) is present only in *Nassodonta*.

Fretter (1941) and deMaintenon (2001) reported the presence of a diverticulum connecting the base of the prostate with the mantle lumen in *Nassarius*, which was found to be present but fused shut in *Tritia obsoleta* (Strong, 2003; as *Ilyanassa obsoleta*). No diverticulum was observed in *Anentome* or *Nassodonta*, although as stated only one reproductively immature male of *Nassodonta* was available for study, nor has one been reported in Dorsaninae or *Buccinanops* (Simone, 1996; Simone & Pastorino, 2014). Consequently, this feature may be a synapormorphy of, or derived within, the Nassariinae.

Based on his study of *Buccinanops* (Simone, 1996), Simone (2011: 209) characterized the oviduct of nassariids as, “…very simple, being little more than a uniformly tubular and glandular structure.” Here, we have found the oviduct to present several elaborations in *Nassodonta* and *Anentome* that are not found in any other nassariids and support the recognition of a new subfamily to unite them. In *Anentome*, the ventral channel of the glandular oviduct continues as a long, tubular, non-glandular vestibule. At its anterior end, it opens to a large muscular, dorsally expanded vagina. Just before its entrance to the vagina, the vestibule dorsally receives the opening of a long, tubular copulatory bursa. The vagina opens via a single, elongate ventral pore to the mantle cavity. The oviduct of *Nassodonta* is built on much the same plan, but presents several unique features. The vestibule receives the opening of a copulatory bursa near its anterior extent but the bursa was found to be vestigial and may be non-functional as no sperm were found within it in histological sections. A large, bulbous, muscular vagina is present, but it and the vestibule open separately to the mantle cavity. It is unclear what the functional significance of this separation may be. In *Tritia* (= *Ilyanassa*; Nassariinae), at the anterior end of the glandular part of the oviduct, a very short, narrow, thin walled vestibule emerges with the female pore at its terminus; a distinct vagina and bursa are both lacking, but a pocket within the anterior capsule gland was hypothesized to function as a bursa (Strong, 2003). In Buccinanopsinae, the tubular vestibule may be thin or thick walled, but again both a bursa and expanded vagina are lacking (Simone, 1996). In Dorsaninae and Nassariinae, the thickened, terminal part of the oviduct (= vestibule) has been reported to function as a bursa (Simone & Pastorino, 2014) and in some nassariines, a distinct bursa has been described (Fretter, 1941; Johansson, 1957; deMaintenon, 2001). Fretter (1941) described the vestibule and the bursa opening to the vagina via separate entrances much like in anentomines, but the vagina was apparently unremarkable, while deMaintenon (2001) described the bursa lying between the female pore and the short vestibule, with the bursa itself opening laterally to the mantle cavity. Given the presence of both a large, expanded vagina and a separate bursa in anentomines, and the positional relationships of the female pore and “bursa” in other nassariids, it seems possible that the bursa of anentomines is a novel acquisition, and the expanded “vagina” has been co-opted to function as a bursa in more derived nassariids. Clearly, the homologies of these structures across the family require further evaluation.

The albumen gland is significantly larger in *Nassodonta* than in *Anentome*, and has invaded the viscera, extending almost to the posterior end of the kidney; the oviduct protrudes into the kidney also in Dorsaninae, but not to a similar extent (Simone & Pastorino, 2014). Ingesting glands lie between the capsule and albumen glands and are connected to the ventral channel via a narrow duct. They are present in Nassariinae (Fretter, 1941; Johansson, 1957; deMaintenon, 2001; Strong, 2003), but are lacking in Anentominae as well as in Dorsaninae and in Buccinanopsinae (Simone, 1996; Simone & Pastorino, 2014). As in other freshwater gastropods, *Anentome* deposits egg capsules on firm substrates and development is nonplanktotrophic; eggs are deposited in clutches of one to four eggs from which crawling juveniles emerge (Coelho, Dinis & Reis, 2013). This reproductive strategy has important implications for limiting dispersal and gene flow, and hence for speciation via isolation by distance.

### Diversification of the Anentominae

Results of the molecular analyses support the recognition of at least four lineages of *Anentome* “*helena*.” All but one are restricted to a single site, with *Anentome* sp. C recovered from two sites in peninsular Malaysia and one in Sumatra. Despite the allopatric distribution of these populations, the modern distribution of this species reflects paleo-drainage connectivity across the Malacca Straits river system, which drained large portions of the west coast of peninsular Malaysia and the east coast of northern Sumatra, northwest to the Andaman Sea during the last glacial maximum (de Bruyn et al., 2013). Connectivity between these populations would have been maintained at least until ∼13,000 years bp when extensive land bridges still existed in this area (Voris, 2000; Sathiamurthy & Voris, 2006). Faunal links across the Straits are well documented, especially among freshwater fishes, and have formed the basis for recognition of the Northern Central Sumatra—Western Malaysia ecoregion (Abell et al., 2008).

While all four lineages received high support (Figs. 3A and 4; PP ≥ 0.97) in the analyses of the mitochondrial and concatenated datasets, specimens identified here as species B from a single site in northern Thailand displayed unusually high Kimura-corrected average pairwise distances for COI and 16S (COI: 0.080; 16S: 0.026–0.045). Compared to the distances within species A, C and D (COI: 0.000–0.012; 16S: 0.000–0.016), this suggests that there may be three lineages represented among the three specimens available for study of species B. Comparison of the shells, however, reveals them to be almost identical, and it is difficult to imagine that these represent three species, particularly as they were all sampled from the same locality. We conservatively interpret this pattern to be indicative of the pronounced mitochondrial structuring that can be found within some terrestrial (Greve et al., 2010; Kotsakiozi et al., 2012) and freshwater (Whelan & Strong, 2016) gastropod populations and which, in the latter, may be maintained through balancing selection (Whelan & Strong, 2016). Additional population-level sampling is necessary to fully characterize the magnitude and geographic scale of mitochondrial structuring among these isolated freshwater populations and to assess the number of discrete lineages.

Regardless of the precise number of molecular lineages, they are all referable to the current broad concept (Brandt, 1974) of *Anentome helena*, formerly *Clea helena*, with its fusiform, prominently ribbed and spirally banded shell. However, none of the populations analyzed here are from Java, so determining which, if any, of these lineages represents true *Anentome helena* is problematic. As stated, all but one of the lineages are restricted to single sites, with one lineage having a relictual distribution reflecting paleo-drainage connectivity across the Malacca Straits in what is today recognized as the Northern Central Sumatra—Western Malaysia ecoregion. Indeed, all other sampled lineages represent distinct paleo-drainages and distinct freshwater ecoregions: *Anentome* sp. A is found in the Lower Salween ecoregion that would have drained west to the Andaman Sea; *Anentome* sp. B is found in the Mekong Delta ecoregion, representing the Mekong paleo-drainage that drained south to the South China Sea; and *Anentome* sp. D is found in the Southern Annam ecoregion which would have drained east to the South China Sea. Java, southeast Sumatra and western Borneo comprise several ecoregions today, but large portions of these islands would have been drained by the East Sunda paleo-drainage, a large river system that flowed south to the Java Sea (Voris, 2000; de Bruyn et al., 2013). Thus, the type specimens of *Anentome helena* would correspond to both a modern ecoregion and a paleo-drainage distinct from any of those represented by the populations included here. Phylogenies of freshwater fishes from Southeast Asia have been found to display a high degree of concordance with paleo-drainage patterns (de Bruyn et al., 2013). Consequently, given the geographic scale of this species complex, the conchological distinctiveness of the lineages, their modern distributions and inferred historical connectivity, it is likely that none of the molecular lineages revealed here represents true *Anentome helena*. This is especially true of the aquarium trade specimens conspecific with species A from Thailand and which bear little conchological resemblance to the types (Fig. 1).

While our analysis has revealed a previously unrecognized radiation within what was formerly recognized as a single species, comprehensively revising the systematics remains a significant challenge. Establishing the identity of *Melania helena* requires topotypic material, but the types are poorly localized simply to “Java.” With the benefit of the insights obtained from the molecular analyses presented herein, it is possible to conclude that the type material likely represents at least two conchologically distinct species given the range of morphological variation (see Fig. 1). Consequently, the prospect that additional members of the *Anentome helena* complex are present on Java is high, or at least historically were present. A further complicating factor is that the freshwaters of Java, as of so many other areas in SE Asia, have been devastated by human-mediated impacts, including those from agriculture, mining, pollution, impoundment and habitat loss (Köhler & Glaubrecht, 2005). A number of freshwater species, including snails and shrimp, are believed to already have gone extinct (Köhler & Glaubrecht, 2005; Marwoto & Isnaningsih, 2012; De Grave et al., 2015). In addition, we require topotypic or near-topotypic material for the other eight nominal species currently in the synonymy of *Anentome* “*helena*,” with type localities stretching from Thailand and Cambodia to Java, a number of which are similarly poorly localized. Consequently, revising the taxonomy of this assemblage would be premature. For the present, we prefer to keep these lineages in open nomenclature pending a thorough systematic revision including broad geographic and population-level sampling. It is probable that the actual diversity of the genus is higher than presently appreciated, although the validity of the other currently recognized species remains to be tested.

### Implications for conservation

As mentioned, assassin snails have recently become a popular commodity in the ornamental pet trade as agents of biocontrol for pest snails and are widely available in aquarium stores and for purchase on the internet. Given their voracious but non-selective appetite for living snails as well as carrion, their introduction and spread constitute a significant threat to native aquatic snail faunas (Mienis, 2011; Bogan & Hanneman, 2013) which are often highly imperiled (Lydeard et al., 2004; Strong et al., 2008; Johnson et al., 2013). Given the record of both deliberate and inadvertent introductions of non-native snails through the aquarium industry (Cowie & Robinson, 2003; Padilla & Williams, 2004; Strecker, Campbell & Olden, 2011), particularly in freshwater, this danger is all too real and already becoming a reality. Recently, Ng et al. (2016b) documented the first non-native establishment of *Anentome* “*helena*,” recorded from Kranji Reservoir in Singapore which is a global hub in the ornamental pet trade.

The discovery that *Anentome* “*helena*” comprises a cryptic complex of at least four species substantially complicates the issue. One sample from the US aquarium industry analyzed here has proven to be the same species as that introduced in Singapore, and based on shell morphology, these appear conspecific with samples in the US Department of Agriculture Animal and Plant Health Inspection Service Plant Protection and Quarantine (USDA APHIS PPQ) reference collection intercepted since May 2009 at several ports of entry (Houston, San Francisco, Los Angeles) from Thailand and Hong Kong. However, at present it is unknown if this species is the source for all specimens marketed under this name. It is also possible that other closely related species of *Anentome* may be marketed indiscriminately as “assassin snails.” More comparative work is necessary to clarify this issue.

Of equal importance is the identity of this species. It is likely none of the species in this complex represent true *Anentome helena* based on comparison with the type material and evidence from paleo-drainage patterns. This question cannot be answered conclusively until DNA sequences of topotypic specimens from Java can be compared with those obtained here. The importance of a solid systematic foundation is evident in other cases where ambiguity about the taxonomy and circumscription of highly invasive species has resulted in enormous confusion, preventing meaningful comparisons of their biology and invasive potential, and hindering management efforts (Hayes et al., 2012; Meyer et al., 2017). In other cases, the impacts of introduced species are more benign, but the resulting confusion may have other unforeseen consequences. For example, misidentified invasive species may be erroneously considered native, narrow range endemics (Ng, Tan & Yeo, 2015). This could direct already strained conservation resources toward undeserving targets. Consequently, resolving basic taxonomic issues is more than just an academic exercise but can have direct economic impacts. Although this research represents a vital first step toward understanding the origins and diversity of this lineage, comprehensive systematic revision of this previously unrecognized species complex is urgently needed to facilitate communication and to manage this emerging threat.

## Supplemental Information

10.7717/peerj.3638/supp-1 Supplemental Information 1 Mitochondrial analysis fasta format.

10.7717/peerj.3638/supp-2 Supplemental Information 2 Nuclear analysis fasta format.

10.7717/peerj.3638/supp-3 Supplemental Information 3 Concatenated analysis fasta format.

## Additional Information and Declarations

### DNA Deposition

The following information was supplied regarding the deposition of DNA sequences: The GenBank accession numbers are provided in Table 1.

### Data Availability

The following information was supplied regarding data availability: The raw data has been supplied as Supplemental Dataset Files.

### New Species Registration

The following information was supplied regarding the registration of a newly described species: Publication LSID: urn:lsid:zoobank.org:pub:C4D12ABC-3DE3-429B-8207-05D549989DD5. LSID for the new subfamily Anentominae: urn:lsid:zoobank.org:pub:C4D12ABC-3DE3-429B-8207-05D549989DD5.
